# Inhibition of VEGFR2 Activation and Its Downstream Signaling to ERK1/2 and Calcium by Thrombospondin-1 (TSP1): *In silico* Investigation

**DOI:** 10.3389/fphys.2017.00048

**Published:** 2017-02-06

**Authors:** Hojjat Bazzazi, Jeffery S. Isenberg, Aleksander S. Popel

**Affiliations:** ^1^Department of Biomedical Engineering, School of Medicine, Johns Hopkins UniversityBaltimore, MD, USA; ^2^Division of Pulmonary, Allergy, and Critical Care Medicine, Department of Medicine, Heart, Lung, Blood, and Vascular Medicine Institute, University of PittsburghPittsburgh, PA, USA

**Keywords:** TSP1, CD47, VEGF, VEGFR2, computational modeling, calcium, ERK1/2

## Abstract

VEGF signaling through VEGFR2 is a central regulator of the angiogenic response. Inhibition of VEGF signaling by the stress-induced matricellular protein TSP1 plays a role in modulating the angiogenic response to VEGF in both health and disease. TSP1 binding to CD47 inhibits VEGFR2 activation. The full implications of this inhibitory interaction are unknown. We developed a detailed rule-based computational model to inquire if TSP1-CD47 signaling through VEGF had downstream effects upon ERK1/2 and calcium. Our Simulations suggest that enhanced degradation of VEGFR2 initiated by the binding of TSP1 to CD47 is sufficient to explain the inhibition of VEGFR2 phosphorylation, calcium elevation, and ERK1/2 activation downstream of VEGF. A complementary mechanism involving the recruitment of phosphatases to the VEGFR2 complex with consequent increase in the rate of receptor dephosphorylation may augment the inhibition of the VEGF signal. The model was then utilized to simulate the effect of inhibiting external TSP1 or the depletion of CD47 as potential therapeutic strategies in restoring VEGF signaling. Results suggest that depleting CD47 is a more efficient strategy in inhibiting the effects of TSP1/CD47 on VEGF signaling. Our results highlight the utility of *in silico* investigations in elucidating and clarifying molecular mechanisms at the intersection of TSP1 and VEGF biology and in differentiating between competing pro-angiogenic therapeutic strategies relevant to peripheral arterial disease (PAD) and wound healing.

## Introduction

VEGF is crucial in normal angiogenesis during embryonic vascular development (Breier et al., 1992; Breier, 2000), wound healing (Bao et al., 2009), and the control of adult vascular permeability and homeostasis (Ku et al., 1993; Lee et al., 2007; Curwen et al., 2008; Lazarus and Keshet, 2011). Under pathological angiogenesis such as cancer, VEGF is also crucial in the development of tumor vasculature and subsequent metastasis (Saharinen et al., 2011). In ocular diseases such as age-related macular degeneration and macular edema, elevated VEGF results in abnormal increase in vascular permeability and aberrant vascular growth in the eye (Miller et al., 2013). In peripheral arterial disease (PAD), the blood flow to the lower extremities is compromised as a result of atherosclerotic occlusion leading to vascular loss. This may partly be attributable to the inhibition of pro-angiogenic signaling and endothelial cell resistance to VEGF (Annex, 2013). An important arm of VEGF signaling is initiated by its binding on endothelial cells to its cognate receptor, vascular endothelial growth factor receptor type 2 (VEGFR2), and the co-receptor neutopilin-1 (NRP1). The signaling cascades downstream of VEGFR2 support endothelial cell survival, proliferation, and motility while suppressing inflammation. VEGF-VEGFR2-NRP1 interaction also leads to the activation of endothelial nitric oxide synthase and the generation of the biogas nitric oxide (NO) with subsequent increase in vascular permeability and vasodilation. Clinically, development of antibodies targeting VEGF as well as kinase inhibitors targeting VEGFR2 have been effective in controlling pathological angiogenesis in diseases characterized by abnormal angiogenesis such as age related macular degeneration, diabetes induced macular edema, and cancer (Rosenfeld et al., 2006; Tah et al., 2015; Ferrara and Adamis, 2016).

Physiological angiogenesis is regulated by endogenous inhibitors with diverse set of mechanisms of inhibition (Folkman, 2004). These largely secreted inhibitors function in the extracellular space by binding and sequestering VEGF or by binding to endothelial cell receptors that initiate signaling cascades that inhibit calcium elevation, NO release, proliferation, and motility. Thrombospondin-1 (TSP1) is a large multi-domain trimeric secreted glycoprotein and among the first identified potent endogenous inhibitors of angiogenesis (Bagavandoss and Wilks, 1990; Good et al., 1990; Taraboletti et al., 1990). The ability of TSP1 to alter cell responses is mediated through interactions with growth factors, cell membrane receptors, and matrix (Isenberg et al., 2009). At nanomolar concentrations, TSP1 can inhibit VEGF signaling by direct binding and sequestering of VEGF (Gupta et al., 1999) or by inducing VEGF/TSP1 complex internalization (Greenaway et al., 2007). At these concentrations, TSP1 can also inhibit the activation of eNOS by AKT via its interaction with the cell surface receptor CD36 (Isenberg et al., 2007). However, at physiological TSP1 concentrations (100–200 pM) endothelial CD47 is a dominant receptor for TSP1. CD47- and TSP1-null animals show enhanced angiogenesis and would healing (Isenberg et al., 2008; Soto-Pantoja et al., 2014). TSP1 interaction with CD47 not only inhibits signaling downstream of NO by inhibiting soluble guanylate cyclase (sGC) and cGMP dependent protein kinase (Isenberg et al., 2006), but it also achieves inhibition of VEGFR2 activation at physiological concentrations (Kaur et al., 2010). Recent evidence suggests that CD47 is pre-associated with VEGFR2 under basal conditions and that TSP1 binding leads to the dissociation of the receptor complex in endothelial cells (Kaur et al., 2010). However, the dissociation between CD47 and VEGFR2 in response to TSP1 is not observed in T cells (Kaur et al., 2014). Although the exact mechanism of action of TSP1 is not yet clear, the CD47-VEGFR2 dissociation is proposed to be the initiating mechanism for the inhibition of VEGFR2 phosphorylation. There is also limited evidence suggesting that VEGFR2 receptors undergo enhanced degradation following the application of TSP1 (Kaur et al., 2011). In this case, TSP1/CD47 interaction acts as a destabilizing factor that reduces receptor degradation with a mechanism not yet understood.

ERK1/2 activation and calcium elevation are important downstream endpoints in VEGF-mediated angiogenic response (Meadows et al., 2001; Faehling et al., 2002; Dellinger and Brekken, 2011; Li et al., 2011; Koch and Claesson-Welsh, 2012). To investigate the consequences of TSP1 binding to CD47 on VEGF-mediated angiogenesis, we concentrate on the effects on ERK1/2 and calcium downstream of VEGFR2. Given the lack of empirical data and the complexity of the signaling processes, we set out here to interrogate potential mechanisms for the inhibition of VEGF/VEGFR2 signaling to ERK1/2 and calcium by TSP1/CD4 interaction utilizing a detailed rule-based model of VEGF signaling to ERK1/2 and calcium incorporating VEGFR2, the co-receptor NRP1, and the detailed dynamics of the receptors. We then included CD47 and TSP1 in this model and carried out exploratory *in silico* simulations to formulate biological mechanisms that would explain and synthesize the available qualitative data. Specifically, we focused on the effects of TSP1 on VEGFR2 receptor dynamics and stability, as well as the empirically suggested inhibition of agonist induced calcium elevation mediated by the inhibition of calcium release-activated calcium channels (CRAC) by TSP1 (Bauer et al., 2010). The model was then applied to quantitatively assess pro-angiogenic therapeutic interventions involving TSP1 and CD47 inhibition with possible utility in pathological conditions such as PAD.

## Materials and methods

The biological rules for receptor interactions and signal transduction to downstream signaling are incorporated in BioNetGen, with the text file (with.bngl extension) given in the Supplementary Material Section. We also included the SBML file associated with the model.

Table S1 contains the list of parameters and their description. Table S2 contains the initial values for the seed species in the model. The rules generate 627 species and 4174 reactions.

Binding of PLCγ to pVEGFR2 and subsequent phosphorylation and dissociation of PLCγ from the receptor is described by a Michaelis-Menten type reaction as follows:

(1) $$\begin{array}{c} \text{pVEGFR}2+\text{PLC}\gamma \to \text{pVEGFR}2+\text{pPLC}\gamma \\ \text{ Rate}=\frac{kp_{\text{PLC}\gamma }[pVEGR2][PLC\gamma ]}{Km_{PLC\gamma /R2}+[PLC\gamma ]} \end{array}$$

pVEGFR2 denotes all the species containing phosphorylated VEGFR2 which is determined by BioNetGen. Using this approach lowers the number of reactions generated by the rules and prevents combinatorial explosion in the model.

Ras activation by S1P is modeled as a Michaelis-Menten type reaction as follows:

(2) $$\begin{array}{c} \text{RasGDP }+\text{ S}1\text{P}\to \text{RasGTP }+\text{ S}1\text{P}\\ \text{ Rate }=\;\frac{k_{\text{S}1\text{P}/\text{Ras}}[\text{S}1\text{P}]}{[S1\text{P}]+Km_{\text{S}1\text{P}/\text{Ras}}} \end{array}$$

with parameters k_S1PRas_ and K_m, S1PRas_ determining the strength of Ras activation by S1P.

Current through the CRAC channels is modeled according to the following equation. This model is a simplified version of the CRAC component of the mathematical model developed by Schmeitz et al. (2013) in T-cells:

(3) $$\begin{array}{c} \frac{dJ_{CRAC}}{dt}=\frac{I_{CRAC}-J_{CRAC}}{\tau_{CRAC}}\\ I_{CRAC}=\frac{{Ī}_{CRAC}K_{CRAC}^{4.2}}{K_{CRAC}^{4.2}+Ca_{ER}^{4.2}} \end{array}$$

The steady-state current (Equation 4) as a function of ER calcium concentration is described by an empirically determined Hill function (Luik et al., 2008).

The output from BioNetGen was saved into C programming language file with MEX functionality which is readable from within MATLAB (the Mathworks Inc., Natick, MA, 2015). The set of ordinary differential equations (ODEs) describing the reaction network was numerically integrated using SUNDIAL numerical solver suite (Hindmarsh et al., 2005). The direct search method implemented in the *patternsearch* function in MATLAB was utilized for parameter fitting.

Western blot images were extracted and analyzed using the software imageJ (Schneider et al., 2012).

Global sensitivity analysis was performed using the partial rank correlation coefficient (PRCC) algorithm described in Marino et al. (2008). The parameter values for sensitivity analysis were randomly chosen from a uniform distribution within a range 0.01 × fitted value ≤ p ≤ 50 × fitted value.

## Results

### Rule-based computational model of VEGF signaling to ERK1/2 incorporating TSP1/CD47 interaction

**Figure 1:**
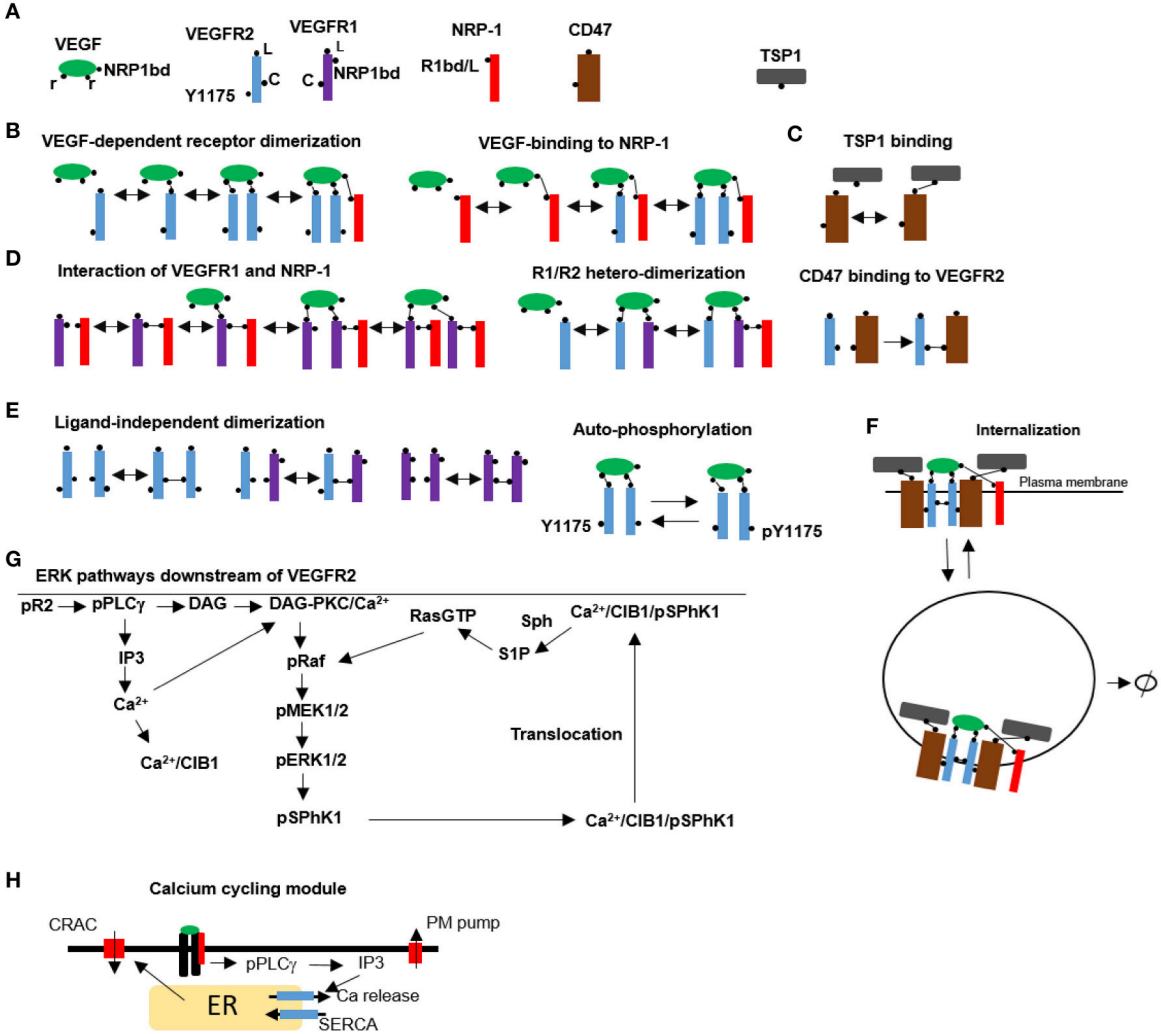
**Rules and pathways for VEGF signaling to calcium and ERK1/2. (A)** Seed species used as input into BioNetGen including VEGF, VEGFR1, VEGFR2/CD47, NRP1, and TSP1. **(B)** Schematic representation of the rules for VEGF-mediated receptor dimerization, VEGF interaction with NRP1, **(C)**. TSP1 binding to CD47 on the cell surface, **(D)**. Ligand-independent VEGFR1 binding to NRP1 and multi-complex formation, VEGF-mediated heterodimer formation between VEGFR1 and VEGFR2. Binding of CD47 to VEGFR2 to form a preassociated complex, **(E)**. Ligand-independent dimer formation resulting in VEGFR2/VEGFR2, VEGFR1/VEGFR1, and VEGFR1/VEGFR2 heterodimers. The rule for autophosphorylation is also shown, **(F)**. Internalization of the complexes that contain VEGF/VEGFR2/VEGFR2 homodimers. **(G)** Signal transduction pathway from the phosphorylated tyrosine to ERK1/2 and calcium incorporating SphK1 feedback and translocation utilizing CIB1, **(H)**. Calcium cycling module incorporating IP3-dependent release from the ER, CRAC, and calcium regulation with plasma membrane and SERCA pumps.

To accurately capture receptor dynamics and signaling to downstream targets, we opted to use a rule-based modeling approach where molecular details of the species and the corresponding rules for the interactions are implemented in a programing environment such as BioNetGen (see the supplementary material for the BioNetGen file) (Faeder et al., 2009; Hlavacek and Faeder, 2009). The program utilizes biological rules to automatically generate the interaction network outputting all the relevant species and chemical reactions. This approach takes into account the combinatorial complexity inherent in multi-domain protein interactions. Another advantage is that the program has a modular structure with parameters, seed species, and reaction rules provided in a single file for easy access and analysis. Figure 1A shows the seed species that are used as input to the model with the corresponding binding and modification sites. VEGF has three binding sites, two for binding to receptors, and a third binding site for binding to NRP1 (Parker et al., 2012). VEGFR2 has three sites: the ligand binding domain to VEGF (L), a ligand-independent coupling site to VEGFR1 or VEGFR2 (C) (Neagoe et al., 2005), and a tyrosine site modifiable by phosphorylation/de-phosphorylation (denoted by Y1175, but it should be interpreted as the lumping of all the relevant phosphorylation modification sites into a single site). CD47 has a binding site for TSP1 and another domain capable of binding and associating with VEGFR2 there is evidence to suggest that CD47 is also associated with signal regulatory protein α [SIRPα and that this interaction maybe important in TSP1 effects in endothelial cells (Yao et al., 2014). Here we assume that CD47/SIRPα are lumped together and modeled as a single agent]. VEGFR1 has a ligand binding site (L), a ligand-independent coupling site to VEGFR1 and VEGFR2 (C), and a binding domain for NRP1 (NRP1bd). NRP1 has a single binding site that is capable of binding competitively to either VEGF or VEGFR1 (R1bd/L) (Fuh et al., 2000). TSP1 has a single binding site for CD47. Rules for VEGF dependent receptor dimerization and VEGF binding to NRP1 with subsequent binding to VEGR2 and dimerization are shown in Figure 1B. The rule for TSP1 binding to CD47 is illustrated in Figure 1C. Figure 1D depicts the rules for the interaction between VEGFR1 and NRP1. The rule for VEGF-mediated hetero-dimerization of VEGFR1 and VEGFR2 is also shown. The rule for the binding between CD47 and VEGFR2 is also drawn. In the absence of ligand, VEGFR1 and VEGFR2 can pre-dimerize (Figure 1E). The rule for VEGFR2 auto-phosphorylation and dephosphorylation is also shown (Figure 1E). Receptor complexes containing VEGF/VEGFR2 heterodimers can undergo endocytosis followed by degradation as illustrated by Figure 1F. CD47 is assumed to undergo recycling and degradation when associated with VEGFR2.

The signaling pathway from phosphorylated VEGFR2 to ERK1/2 and calcium is depicted in Figure 1G. The pathway was constructed by reviewing the literature evidence for the components of VEGF mediated ERK1/2 activation and the relevant pathway information contained in Reactome database (Milacic et al., 2012; Croft et al., 2014). ERK1/2 activation downstream of VEGFR2 is dependent on PKC and the positive feedback resulting from the phosphorylation of SphK1 by pERK1/2, and the subsequent translocation of phosphorylated SphK1 to the plasma membrane by calcium and integrin binding protein 1 (CIB1) (Kolch et al., 1993; Shu et al., 2002; Pitson et al., 2003; Jarman et al., 2010). CIB1 contains a myristoyl switch that is activated by the binding of calcium. Once in the plasma membrane, SphK1 phosphorylates and generates S1P which then activates Ras. While the exact mechanism for the activation of Ras by S1P is not known, it is thought to involve the inhibition of a GTPase activating protein.

One distinguishing feature of the model here is the incorporation of a detailed calcium module downstream of VEGFR2 that includes release from the endoplasmic reticulum (ER) via the IP3 sensitive receptors and calcium regulation involving plasma membrane and SERCA pumps. CRAC channels are also a prominent hallmark of the calcium cycling module (Figure 1H) (see methods for the description of the CRAC channel model). The model for calcium dynamics including calcium buffering in cytoplasm and the ER is similar to the model developed in Silva et al. (2007). The computational model is parameterized by fitting the model simulations to a consistent set of published experimental data on VEGFR2 receptor dynamics, dynamics of various phosphorylated species and the dose-response curves for the activation of VEGFR2 and ERK1/2. The model is simultaneously fitted to the available data to estimate and constrain the parameters. Global sensitivity analysis is also performed to isolate the most significant parameters affecting model dynamics. The list of the parameter values along with their description is included in Table S1 as part of the Supplementary Material. Receptor levels are shown in fraction total for ease of comparison with the experimental measurements.

### Model simulations and sensitivity analysis

**Figure 2:**
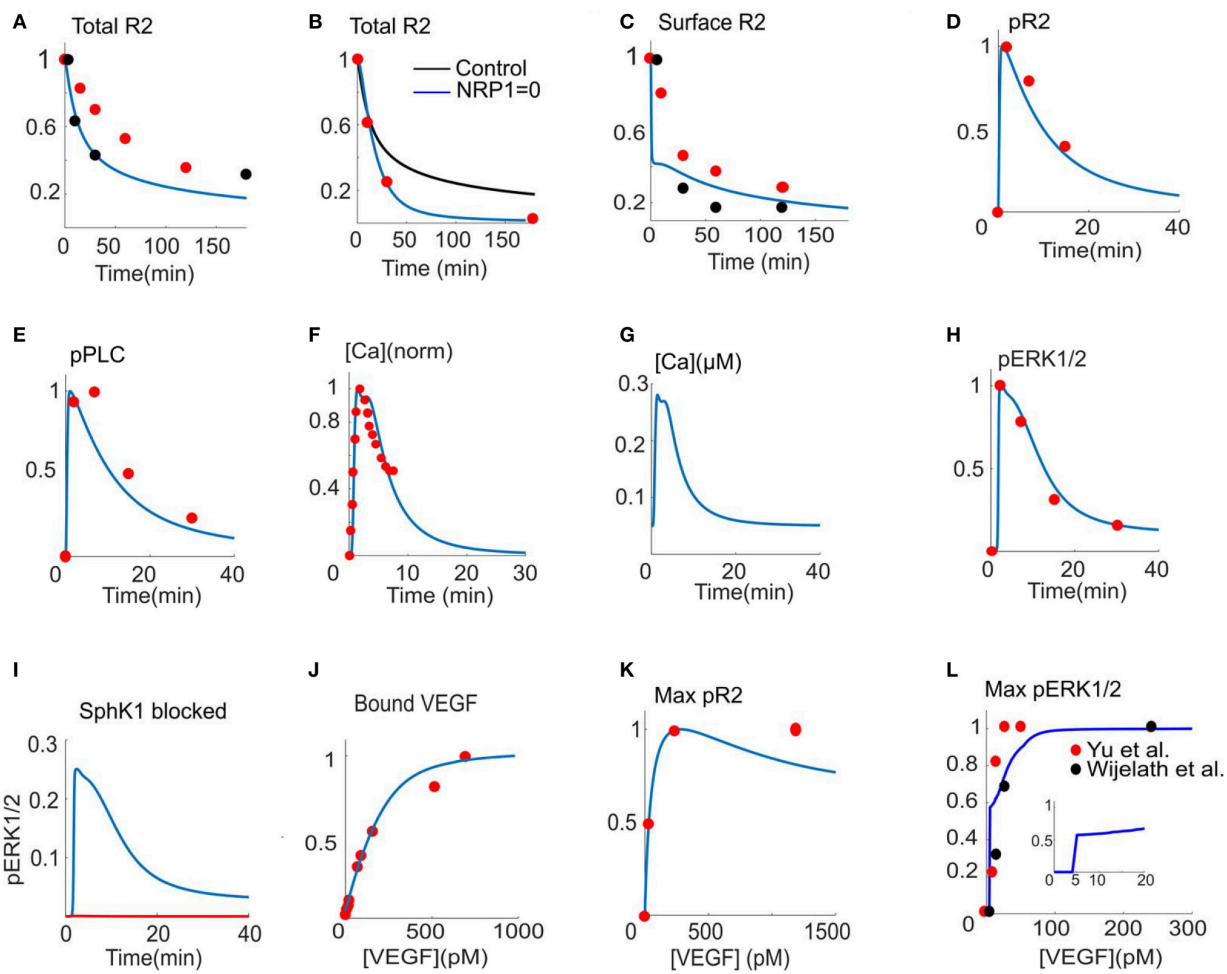
**Model parameterization. (A)** Total VEGFR2 (R2) computed as fraction total versus time fitted to two sets of experimental data from Bruns et al. (2010) and Ballmer-Hofer et al. (2011) (red and black circles), **(B)**. R2 versus time with (blue) and without (black) NRP1 fitted to the data in Ballmer-Hofer et al. (2011) (red circles), **(C)**. Surface level of VEGFR2 constrained with two sets of experimental data from Ewan et al. (2006) and Bruns et al. (2010) (red and black circles). **(D)** Fraction of phosphorylated receptors (pR2) is fitted to the experimental data in Chabot et al. (2009) (red circles). **(E)** Phosphorylated PLCγ from the model fitted to the data in Chabot et al. (2009) (red circles). **(F)** Normalized calcium transient from the model fitted to the normalized experimental data in Li et al. (2011) (red circles). **(G)** Raw calcium trace corresponding to the normalized transient in **(F)**. **(H)** pERK1/2 fit to the data in Chabot et al. (2009) (red circles). **(I)** SphK1 inhibition abolishes pERK1/1 consistent with the data in Shu et al. (2002) (red versus blue curves). **(J)** Binding of VEGF to the binding sites on the cell surface versus VEGF concentration (Bikfalvi et al., 1991), **(K)**. Dose response curve for pR2 is fitted to the experimental data in Whitaker et al. (2001) (red circles), **(L)**. Dose response curve for the activation of ERK1/2 is fitted to two sets of data from Wijelath et al. (2006) and Yu et al. (2015) (red and black circles). The inset indicates sudden jump in pERK1/2 as VEGF in increased above threshold.

In Figure 2A, the total VEGFR2 level from the model (solid blue) is fitted to the two sets of experimental data (red and black circles) (Bruns et al., 2010; Ballmer-Hofer et al., 2011). After 180 min of continuous exposure to VEGF, over 80% of the receptors are degraded. Empirical data suggest that NRP1 stabilizes VEGFR2 at the cell surface and that in the absence of NRP1, VEGFR2 recycling to the plasma membrane is seriously hindered. As shown in Figure 2B, the model is constrained by simulating the effect of the absence of NRP1 by fitting the simulated VEGFR2 level from the model to the data (red circles) (Ballmer-Hofer et al., 2011). The control curve (black) shows that after 180 min of VEGF exposure, the cell retains 20% of the receptors. In the absence of NRP1, however, all the receptors are degraded (blue). The fitted values of the parameters for internalization, membrane recycling, and degradation reveal that in the absence of NRP1, the recycling rate to the membrane from the endosomal compartment is substantially decreased (0.756 s^−1^ vs. 1.24 × 10^−3^ s^−1^). This reduction in recycling rate is sufficient to explain the rapid decay of total cellular receptor levels in the absence of NRP1. In fact, it is the combined effects of internalization, recycling, and degradation that determine the rapid decay of VEGFR2 cellular level (Figure 2B). The surface fraction of VEGFR2 is fitted to two sets of experimental data gathered by flow cytometry (red circles) (Ewan et al., 2006) or Western blot (red circles) (Figure 2C) (Bruns et al., 2010). Surface receptors undergo rapid internalization upon continuous VEGF exposure. The model demonstrates rapid VEGFR2 internalization within the first 5 min (60% internalized in ~5 min). The fraction of phosphorylated VEGFR2 (pR2) and phosphorylated PLCγ are fitted to the experimental data (Chabot et al., 2009) (red circles) as shown in Figures 2D,E. To determine the values of the relevant parameters governing calcium dynamics, the normalized calcium concentration in the cytoplasm (Figure 2F, blue) is fitted to the normalized experimental data (Figure 2F, red circles) (Li et al., 2011). The raw trace is shown in Figure 2G indicating a transient amplitude of ~250 nM consistent with experimental measurements (Faehling et al., 2002). Figure 2H shows the ERK1/2 activation signal fitted to the data from human umbilical vein endothelial cells (HUVEC) (Chabot et al., 2009). The model is constrained by the experimental evidence that blocking SphK1 inhibits ERK1/2 activation (Figure 2I) (Shu et al., 2002). The parameters for VEGF binding to the receptors on the cell surface are constrained by the experimental data for VEGF binding to the available binding sites on the cell (Bikfalvi et al., 1991) (Figure 2J). The dose-response data for phosphorylated receptors are also utilized (Figure 2K) (Whitaker et al., 2001). To constrain the dose-response of ERK1/2, we utilized two sets of data in HUVEC (Wijelath et al., 2006) (black circles) and pulmonary arterial endothelial cells (PAEC) (Yu et al., 2015) (red circles). Constraining the model revealed a surprising switch-like behavior in ERK1/2 activation occurring at ~5 pM VEGF, implying the existence of threshold in VEGF activation of ERK1/2 at 5 pM. The plot in the inset illustrates this sudden jump in pERK1/2 signal at 5 pM VEGF. Another interesting emergent feature of the model is the prediction that the activation of VEGFR2 at the surface is transient, decaying to the baseline in less than 10 min, while the main signaling occurs from the internalized receptors, sustained for 30 min (Figures S1A,B). This is consistent with current experimental and computational modeling evidence arguing for the critical role of endocytosis in shaping and regulating VEGFR2 signaling (Zhang et al., 2013; Clegg and Mac Gabhann, 2015). Figures S1C–F also shows the predicted traces for the activation of PKC, Ras, Raf, and the dose-response curve for RasGTP, with the same threshold behavior as ERK1/2.

To identify sensitive parameters involved in VEGFR2 activation, we carried out global sensitivity analysis summarized in Figures S1G,H. Not surprisingly, the total VEGFR2 level is the most sensitive parameter affecting pVEGFR2 with positive correlation. The next in the ranking are parameters describing the interaction between VEGFR2 and NRP1. The top ranking negatively correlated parameter is the degradation rate of VEGFR2. The next on the list are parameters determining the interaction of VEGFR2 and NRP1 (see Table S1 for the detailed description of the parameters along with their values). Figure S2 summarizes the result of global sensitivity analysis identifying positive and negatively correlated parameters affecting ERK1/2 activation.

As discussed previously, there is empirical evidence for the enhanced degradation of VEGFR2 following TSP1 application (Kaur et al., 2011). In the next section, we utilize the model to perform predictive simulations to test whether the enhanced degradation of VEGFR2 is sufficient to explain the TSP1 inhibitory effects on VEGF signaling to ERK1/2 and calcium.

### Can enhanced VEGFR2 degradation initiated by TSP1 binding to CD47 explain the inhibitory effects of TSP1?

**Figure 3:**
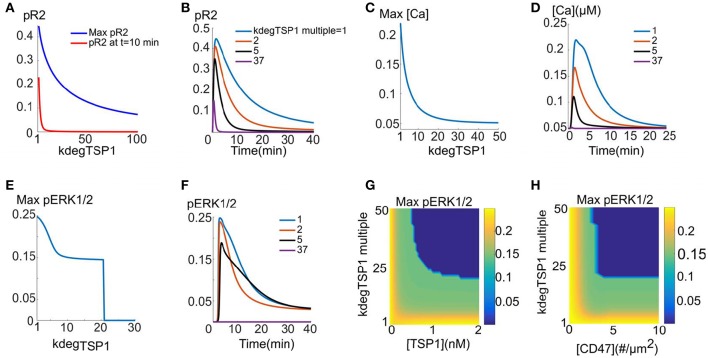
**TSP1 inhibition of VEGFR2 signaling with enhanced VEGFR2 degradation**. Two nanometer TSP1 is added for 10 min followed by 40 min of 50 ng/ml VEGF. **(A–F)** pR2 **(A,B)**, calcium **(C,D)**, and pERK1/2 **(E,F)** response as VEGFR2 degradation is increased. ERK1/2 exhibits threshold behavior in response to receptor degradation rate **(E)**. **(G)** Maximum pERK1/2 as a function of TSP1 concentration and the fold-change in VEGFR2 degradation in the presence of TSP1. The dark blue region is the parameter regime where no ERK1/2 activation is observed, **(H)**. Maximum ERK1/2 as a function of CD47 level [# (number) per surface area] and the fold-change in VEGFR2 degradation with TSP1 present.

We simulate the direct effect of TSP1 by assuming that CD47 binding to TSP1 leads to accelerated VEGFR2 degradation. It is assumed that TSP1/CD47/VEGFR2 undergo internalization together and either recycle back to the membrane or degrade with a higher degradation rate (shown schematically in Figure 1F). In cells devoid of C47, VEGFR2 signaling is not affected by TSP1. This implies that TSP1/CD47 interaction is necessary for VEGFR2 degradation. For the simulations here, cells are exposed to 2 nM TSP1 for 10 min, followed by 40 min exposure to 50 ng/ml VEGF. This is similar to the experimental protocol in Kaur et al. (2010). According to Figure 3A, maximum fractional pR2 and pR2 at 10 min decline rapidly as the degradation rate of VEGFR2 is increased. Indeed, a 5-fold increase in degradation rate is sufficient to essentially abolish the plateau phase of the pR2 signal (Figure 3A, red). Figure 3B demonstrates this more clearly by showing pR2 traces for different values of the degradation rate (indicated in the legend as fold changes). A 5-fold increase in the degradation rate is predicted to be sufficient to significantly decrease the activation of VEGFR2 (blue versus black traces). Figures 3C,D demonstrates that the enhanced degradation mechanism is very effective in inhibiting the calcium transient generated downstream of VEGFR2. ERK1/2 activation is robust until a critical value of the degradation rate is reached as shown in Figure 3E (~21-fold increase relative to the base value, or 0.03 s^−1^). Samples traces for four different degradation rates are also shown in Figure 3F. Figure 3G investigates the effect of enhanced degradation of VEGFR2 and external TSP1 concentration on maximum pERK1/2 signal. Accordingly, if VEGFR2 degradation is accelerated by 50-fold, at least 0.52 nM TSP1 concentration is required for the inhibition of ERK1/2 signal. This is consistent with empirical data suggesting that TSP1 inhibits VEGFR2 signaling at concentrations above 0.6 nM (Kaur et al., 2010). Figure 3G further predicts that ~33-fold increase in VEGFR2 degradation is required for the inhibition of VEGFR2 signaling by 0.6 nM TSP1.

The effect of VEGFR degradation and CD47 surface levels on ERK1/2 activation is investigated in Figure 3H. TSP1 concentration is 2 nM for these simulations. The decline in the effect of TSP1 is very steep as CD47 levels are reduced from 5 receptors/μm^2^ (7000 receptors per cell) to 2.2 receptors/μm^2^ (3100 receptors per cell). Figure 3H further predicts that increasing CD47 levels beyond 5 receptors/μm^2^ does not affect TSP1 inhibition of VEGFR2 signaling.

Overall, the results here demonstrate that the enhanced degradation of VEGFR2 by TSP1/CD47 interaction is a viable mechanism to explain the global shutdown of VEGFR2 signaling. The dependence on TSP1 concentration is also consistent with the experimental data indicating the viability of the enhanced degradation mechanism.

### Phosphatase recruitment to VEGFR2 by TSP1/CD47 interaction may augment the inhibitory effects of TSP1 on VEGF signaling

**Figure 4:**
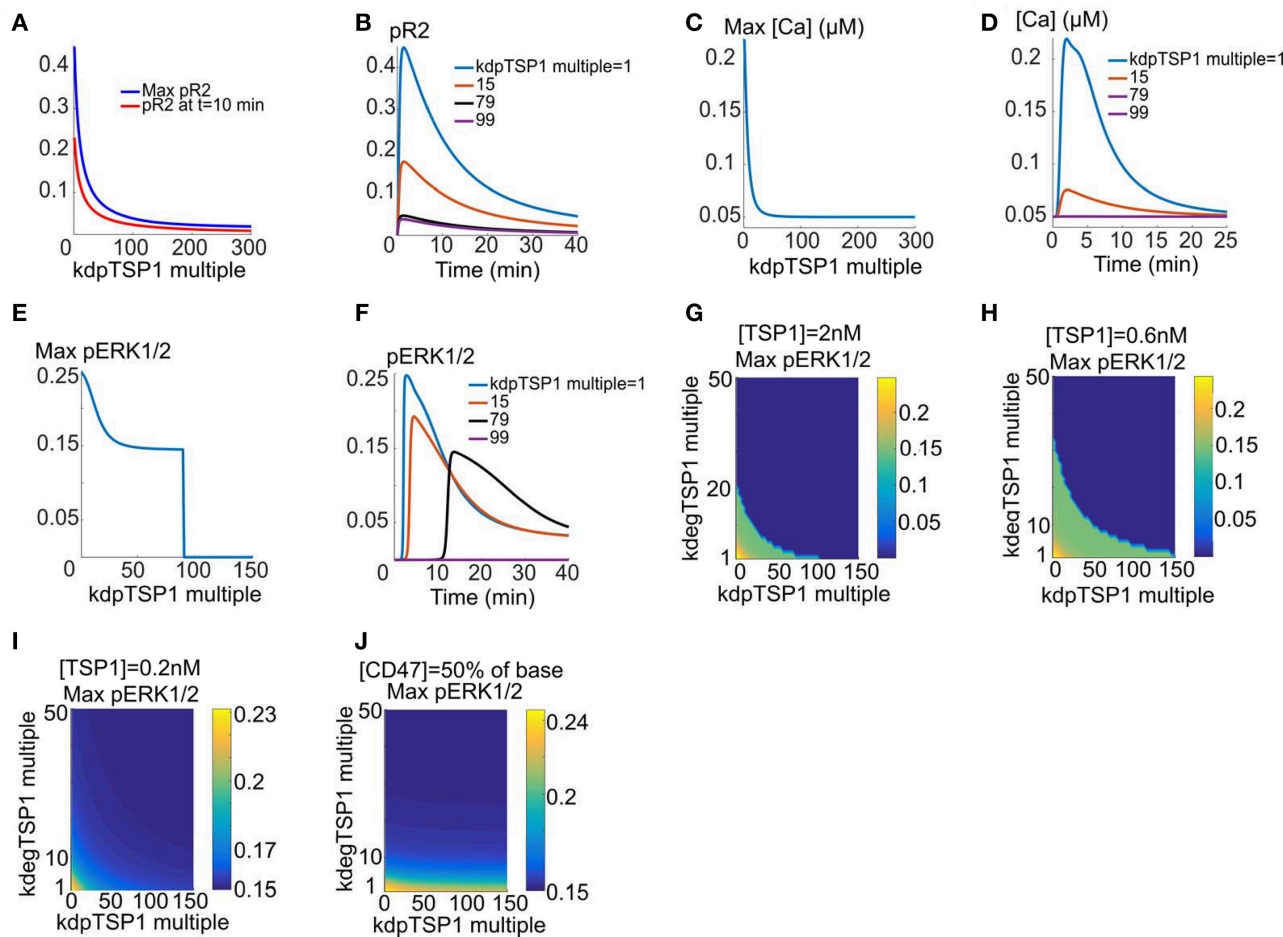
**Enhanced dephosphorylation of VEGFR2 via TSP1-mediated phosphatase recruitment. (A,B)** Max pR2 and pR2 at 10 min vs. the fold-change in VEGFR2 dephosphorylation rate for receptors containing TSP1 with sample traces, **(C,D)** Max [Ca] as a function of the fold decrease in the rate of receptor dephosphorylation and sample traces, **(E,F)** Maximum pERK1/2 vs. fold-change in dephosphorylation rate and sample traces showing the threshold behavior, **(G–I)** Max pERK1/2 as a function of enhanced VEGFR2 degradation (shown as fold-change relative to control with no TSP1) and the fold-change in receptor dephosphorylation rate for 2, 0.6, and 0.2 nM TSP. This identifys the parameter regime for effective ERK1/2 inhibition (dark blue regions). there is no inhibition with [TSP1] = 0.2 nM **(I)**. **(J)** Max pERK1/2 as a function degradation and dephosphoryation rates with 50% reduction in CD47 levels and 2 nM TSP1.

We next consider the hypothesis that TSP1 might insert some of its inhibitory effects on VEGFR2 by recruiting phosphatases to the receptor complex. For the simulations here the cells are exposed to 2 nM TSP1 for 10 min, followed by the addition of VEGF for 40 min. It is assumed that the increase in dephosphorylation rate is the sole mechanism. As shown in Figure 4A, maximum fractional pR2 and pR2 at 10 min decline rapidly as the dephosphorylation rate (induced by TSP1/CD47 binding) increases. Sample traces are shown in Figure 4B. As shown by the figure, ~80-fold increase in VEGFR2 dephosphorylation rate is sufficient to essentially collapse the pR2 signal (blue versus black trace). Enhanced dephosphorylation is also very effective in shutting down calcium elevation downstream of VEGFR2 as demonstrated by Figures 4C,D. To block ERK1/2 activation the model predicts at least ~90 fold increase in dephosphorylation rate at 2 nM TSP1 (Figure 4E). Sample pERK1/2 traces are shown in Figure 4F. Increasing dephosphorylation delays ERK1/2 activation until the threshold dephosphorylation value is reached beyond which there is no pERK1/2 signal (flat purple trace). Figures 4G,H summarize the effects of combined enhanced degradation and phosphatase recruitment on maximum pERK1/2 at two different TSP1 concentrations shown experimentally to inhibit VEGFR2. The surface plots demonstrate that the two mechanism can work in tandem to inhibit VEGFR2 signaling to ERK1/2. For example, 10-fold increase in VEGFR2 degradation and 51-fold increase in VEGFR2 dephosphorylation rate is predicted to be sufficient to inhibit ERK1/2 activation at 0.6 and 2 nM TSP1. Interestingly, consistent with experimental data, 0.2 nM TSP1 is not effective at inhibiting VEGFR2 signaling within a wide range of degradation and dephosphorylation rates (Figure 4I). Further, 50% reduction in cellular CD47 makes cells resistant to TSP1 effects (Figure 4J). In all, these simulations synthesize the available experimental data on the inhibitory effect of TSP1 on VEGF signaling and demonstrate quantitatively that enhanced degradation of VEGFR2 coupled with phosphatase recruitment to the receptor by TSP1 are effective mechanisms in shutting down VEGF signaling at physiological concentrations of TSP1.

Next, we consider a different layer of TSP1 effects involving the experimentally suggested alteration of calcium signaling by TSP1 (Bauer et al., 2010) and the consequent effect on ERK1/2 dynamics.

### TSP1 inhibition of CRAC channels affects calcium elevation and ERK1/2 dynamics

**Figure 5:**
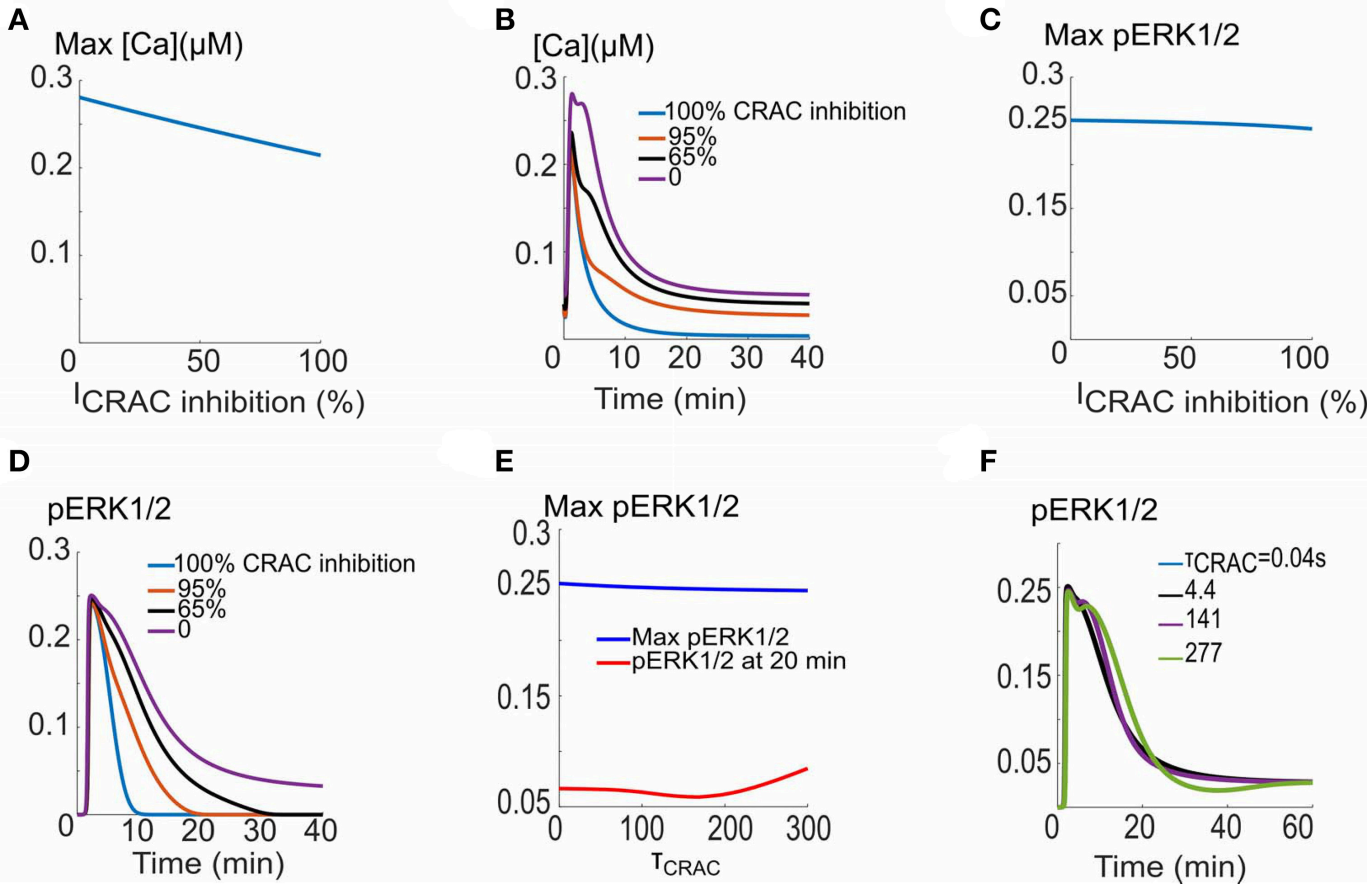
**CRAC channel inhibition and VEGF signaling to ERK1/2 and calcium. (A)** Max Ca versus CRAC channel amplitude, **(B)**. Ca^2+^ traces for different CRAC channel current values. **(C)** Max pERK1/2 vs. current showing no appreciable effect. **(D)** pERK1/2 traces showing prolongation of pERK1/2 signal in response to increase in the current. **(E)** Max pERK1/2 and pERK (at 10 min) as a function of the time constant for the activation of the CRAC current. **(F)** pERK1/2 traces for five different values of the time constant.

Recent experimental evidence suggests that TSP1 inhibits the ionomycin-induced calcium elevation (Bauer et al., 2010). This inhibitory effect may be attributed to the inhibition of CRAC channels by TSP1 with a mechanism not yet understood. The consequences of inhibiting CRAC current amplitude and the activation time constant are studied in Figure 5. As shown in Figure 5A, the calcium transient amplitude does not change appreciably as the current amplitude is inhibited from its base value of 1.74 × 10^4^ μM/s. Sample calcium transients are shown in Figure 5B predicting profound effects on the duration and plateau phase of the calcium transients. The inhibition of CRAC current is also not predicted to affect the maximum of ERK1/2 activation signal (Figure 5C), but significantly influences the plateau phase and duration of pERK1/2 (Figure 5D). Interestingly, the change in activation time constant of the current through the CRAC channels does not affect ERK1/2 activation as illustrated by Figures 5E,F. The simulations demonstrate that within the context of the model here, the hypothesized TSP1 inhibition of CRAC current amplitude, but not current activation dynamics, is capable of profoundly impacting calcium transient and ERK1/2 activation signal morphology.

### Targeting TSP1 and CD47 as potential strategies in restoring VEGFR2 signaling

**Figure 6:**
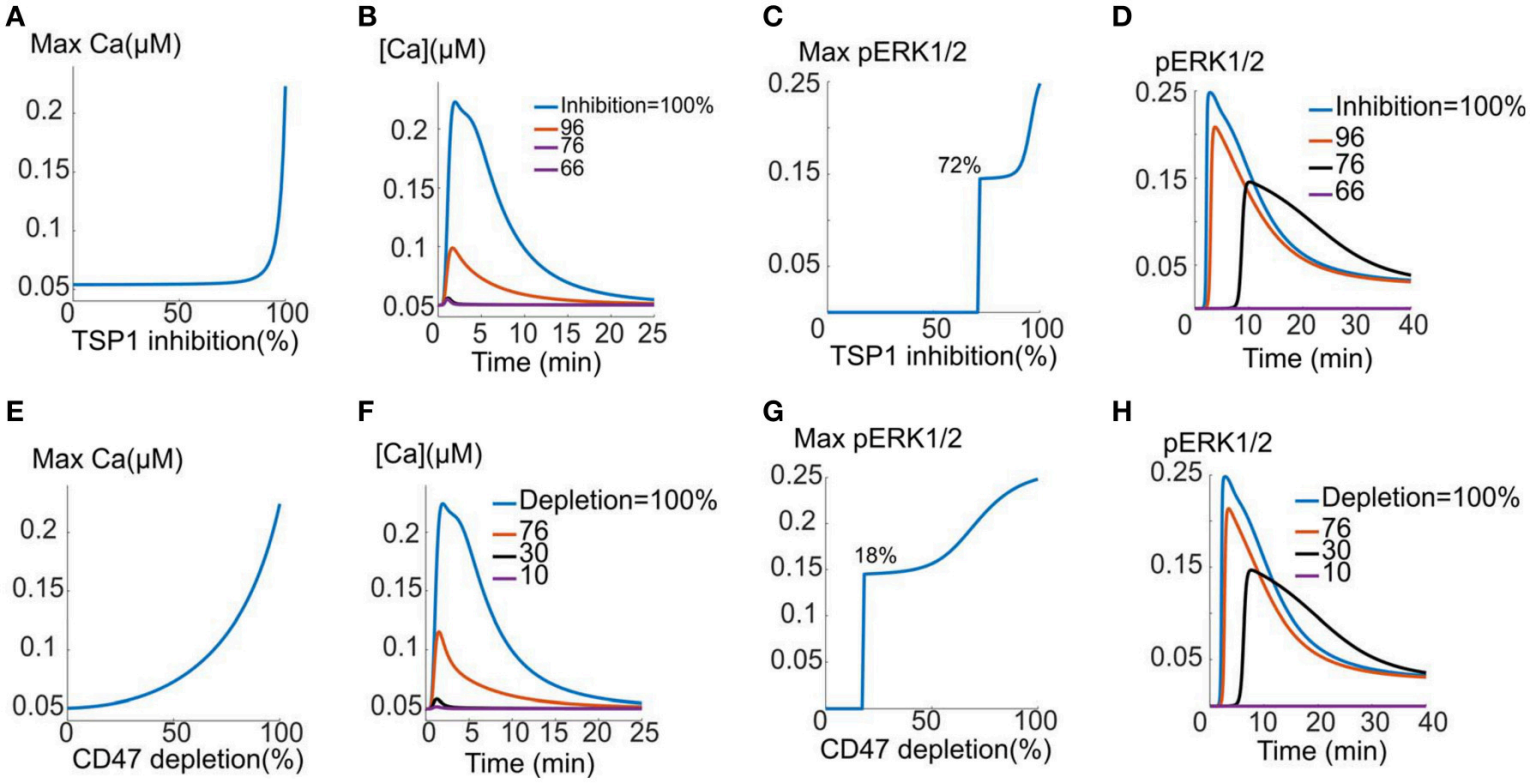
**TSP1 and CD47 inhibition and the restoration of VEGF signaling. (A–D)** TSP1 inhibition and the recovery of the calcium **(A,B)** and pERK1/2 signal indicating 72% inhibition threshold for effective recovery **(C,D)**. TSP1 inhibition involves 10-fold increase in VEGFR2 degradation and 51-fold increase in dephosphorylation relative to the case with no TSP1. **(E–H)** VEGF signal recovery by CD47 depletion. **(E,F)** intracellular calcium recovery as CD47 is depleted along with sample traces, **(G,H)** ERK1/2 activation is restored when 18% of CD47 is depleted. Sample traces are also shown.

Considering the function of TSP1 as a potent inhibitor of VEGF signaling to calcium and ERK1/2 activation, here we carry out proof-of-principle simulations to demonstrate the effectiveness of TSP1/CD47 inhibition as a strategy for restoring VEGF signaling. The effect of TSP1 inhibition (such as the application of anti-TSP1 antibody, inhibition of TSP1 secretion, or siRNA against TSP1 transcript) is modeled in a simple manner by lowering the concentration of TSP1 and monitoring the recovery of calcium and pERK1/2 signals. We consider a mixed mechanism involving enhanced VEGFR2 degradation (10-fold) and increased VEGFR2 dephosphorylation (51-fold). These values are consistent with the two-parameter scan in Figures 4G,H. The inhibition of CD47 is modeled by depleting CD47 (e.g., by anti-CD47 antibody or the use of morpholinos) and monitoring calcium and pERK1/2 signals. It is further assumed that 2 nM TSP1 and 50 ng/ml VEGF are simultaneously added. TSP1 is capable of inhibiting VEGF signaling when added simultaneously with VEGF as demonstrated by Figures S3A–D for enhanced degradation mechanism and Figures 6E–H for increased dephosphorylation). As demonstrated in Figures 6A–D for TSP1 inhibition, both calcium and pERK1/2 signals are eventually restored by sufficiently high TSP1 inhibition. Note that calcium signal is unresponsive to TSP1 inhibition until at least 80% inhibition is achieved. Sample calcium transients are shown in Figure 6B, clearly demonstrating calcium recovery in response to TSP1 inhibition. Similarly, as shown in Figure 6C, inhibiting TSP1 restores ERK1/2 activation and that threshold for ERK1/2 signal recovery is ~72% inhibition. Sample pERK1/2 traces are also included in Figure 6D showing the recovery of the ERK1/2 signal as TSP1 is inhibited. The high TSP1 inhibition threshold (80% for calcium and 72% for ERK) implies that TSP1 inhibition might be challenging as a therapeutic strategy as it would require sustained and high TSP1 inhibition, highlighting the need for effective drug delivery technology capable of achieving above-threshold TSP1 inhibition *in vivo*.

The effect of depleting CD47 as a therapeutic strategy is summarized in Figures 6E–H. Note the stark contrast between calcium recovery curves in Figures 6E,A. According to Figure 6E, calcium signal responds readily to CD47 depletion and the recovery is rapid after 50% CD47 depletion. Sample traces are shown in Figure 6F clearly showing that calcium signaling is restored by CD47 inhibition and that CD47 inhibition is more effective that TSP1 inhibition in restoring the calcium transient. ERK1/2 activation is restored following depletion of only 18% of CD47, once again much more effective than TSP1 inhibition (Figure 6G vs. Figure 6C). Sample pERK1/2 traces are shown in Figure 6H demonstrating the recovery of the ERK1/2 activation signal.

The simulations demonstrate that CD47 depletion may be a more effective strategy than targeting TSP1 in restoring VEGF signaling to calcium and ERK1/2. CD47 depletion is particularly effective in restoring ERK1/2 activation downstream of VEGFR2.

## Discussion

Inhibition of VEGF signaling is a hallmark of anti-angiogenic function of TSP1. Given the scarcity of experimental data elucidating the precise inhibitory mechanisms, we attempted at undertaking an *in silico* approach to computationally investigate potential mechanisms of inhibition. The model incorporated detailed molecular mechanisms using a rule-based model for receptor-receptor interaction and a coarse-grained model for signaling to calcium and ERK1/2 from the activated receptors. The inclusion of detailed calcium cycling module in the model allowed us to study the role of TSP1 in inhibiting calcium signaling and the potential crosstalk with ERK1/2 activation. Fluorescene resonance energy transfer based (FRET-based) experiments in endothelial cells had shown the preassociation of VEGFR2 with CD47 in endothelial and T cells (Kaur et al., 2010, 2014). We hypothesized that TSP1 binding to CD47 might enhance VEGFR2 degradation. Our simulations predicted that this enhanced-degradation scenario is indeed capable of explaining TSP1 effects on VEGFR2 phosphorylation, calcium elevation, and ERK1/2 activation. This TSP1/CD47 dependent enhanced degradation was consistent with the experimental data indicating that the knock-down of CD47 protected against TSP1 inhibition of VEGFR2 (Kaur et al., 2010).

An interesting prediction of the model was that the effect on ERK1/2 activation is switch-like, implying that ERK1/2 activation is robust until a threshold value of the degradation rate is reached. This result is consistent with recent data in cancer cells demonstrating that repression of TSP1 expression is a critical step in establishing the angiogenic switch in tumor cells, the transformation from avascular to vascularized tumors (Watnick et al., 2015). Our simulations corroborate the existence of a switch-like mechanism in TSP1 inhibition of VEGFR2 signaling. Moreover, simulations show that VEGFR2 phosphorylation was more sensitive to TSP1-induced enhanced receptor degradation than ERK1/2 activation. ERK1/2 activation was robust until the degradation rate was increased by over 21-fold (see Figures 3E,F), while VEGFR2 phosphorylation was drastically inhibited by only a 5-fold increase in the degradation rate (Figures 3A,B). This predicted differential sensitivity of the model to TSP1 suggests that simultaneous assaying for ERK1/2 activation and VEGFR2 phosphorylation maybe important in experimental studies aiming to investigate the mechanism of TSP1 action on VEGF signaling. By itself, VEGFR2 response to TSP1 is not a straightforward linear predictor of downstream ERK1/2 effects.

Another mechanism considered here was the increase in VEGFR2 dephosphorylation by TSP1-induced recruitment of phosphatases to VEGFR2/CD47 complex. Simulations demonstrated that the two inhibitory mechanisms may work together to effectively inhibit VEGF signaling.

Simulations here also proposed the possibility of inhibiting TSP1 or CD47 as potential interventions for restoring VEGF-mediated angiogenic response. The model showed that sequestering TSP1 effectively normalized calcium signaling and ERK1/2 activation, albeit requiring at least 80% inhibition. This high threshold for inhibition might preclude the *in vivo* applicability of this strategy in restoring angiogenesis in diseases such as PAD. In related simulations, the computer model predicted that CD47 depletion is more efficient at restoring calcium and ERK1/2 activation (with lower hysteresis). Indeed, only 18% depletion of CD47 was needed to restore ERK1/2 activation signal compared with 72% TSP1 inhibition. The results highlight the important role of efficient and sustained therapeutic agent delivery to affected tissues to ensure above-threshold inhibition of TSP1 and CD47. They further suggest that CD47 depletion should be considered as an initial approach in pro-angiogenic therapeutic intervention. It is plausible that different endothelial cell types may exhibit differential inhibition thresholds to TSP1 and CD47 which would further necessitate the need for sustained delivery of anti-TSP1 and anti-CD47 agents.

Further complicating anti-TSP1 therapy, platelets contain significant pre-formed TSP1 that are a depot source of protein. Thus, targeting CD47 is an alternative strategy expected to be more efficient at preserving pro-angiogenic signals. Additionally, VEGF can also signal via NO while CD47 can interact with cell membrane SIRPα (Yao et al., 2014). Analysis of TSP1-VEGF signaling in relation to these additional components will be important in future extensions of the current model.

## Author contributions

AP conceived and coordinated the study. HB and AP designed the computational model, HB implemented the model in BioNetGen and performed computational analysis. HB and AP wrote the paper. JI contributed to the conceptual understanding and precise definition of the problem and analysis of the results and contributed to the writing of the manuscript. All authors reviewed the results and approved the final version of the manuscript.

### Conflict of interest statement

JI serves as chair of the scientific advisory boards of Radiation Control Technologies, Inc. (Jersey City, NJ) and has equity interest in the same and in Tioma Therapeutics (St. Louis, MO). The other authors declare that the research was conducted in the absence of any commercial or financial relationships that could be construed as a potential conflict of interest.
