# Corrigendum: Inhibition of VEGFR2 Activation and Its Downstream Signaling to ERK1/2 and Calcium by Thrombospondin-1 (TSP1): *In silico* Investigation

**DOI:** 10.3389/fphys.2017.00147

**Published:** 2017-03-07

**Authors:** Hojjat Bazzazi, Jeffrey S. Isenberg, Aleksander S. Popel

**Affiliations:** ^1^Department of Biomedical Engineering, School of Medicine, Johns Hopkins UniversityBaltimore, MD, USA; ^2^Division of Pulmonary, Allergy, and Critical Care Medicine, Department of Medicine, Heart, Lung, Blood, and Vascular Medicine Institute, University of PittsburghPittsburgh, PA, USA

**Keywords:** TSP1, CD47, VEGF, VEGFR2, computational modeling, calcium, ERK1/2

In the original article, there was a mistake in the legend for Figure 3 as published.

“TSP1 inhibition of VEGFR2 signaling with enhanced VEGFR2 degradation. Two nanometer TSP1 is added for 10 min followed by 40 min of 50 ng/ml VEGF…” The correct legend appears below. The authors apologize for this error and state that this does not change the scientific conclusions of the article in any way.

“TSP1 inhibition of VEGFR2 signaling with enhanced VEGFR2 degradation. 2 nM TSP1 is added for 10 min followed by 40 min of 50 ng/ml VEGF…”

**Incorrect Author Name**

An author name was incorrectly spelled as **Jeffery S. Isenberg**. The correct spelling is **Jeffrey S. Isenberg**. The authors apologize for this error and state that this does not change the scientific conclusions of the article in any way.

**Text Correction**

In the original article, there was an error on the **Introduction** section page 2, paragraph 3:

“…we set out here to interrogate potential mechanisms for the inhibition of VEGF/VEGFR2 signaling to ERK1/2 and calcium by **TSP1/CD4** interaction utilizing a detailed rule-based model of VEGF signaling to ERK1/2 and calcium…”

A correction has been made to **Introduction**, paragraph 3:

“…we set out here to interrogate potential mechanisms for the inhibition of VEGF/VEGFR2 signaling to ERK1/2 and calcium by **TSP1/CD47** interaction utilizing a detailed rule-based model of VEGF signaling to ERK1/2 and calcium…”

The authors apologize for this error and state that this does not change the scientific conclusions of the article in any way.

In the original article, there was an error on the **Conflict of Interest Statement**:

“JI serves as chair of the scientific advisory **boards** of Radiation Control Technologies, Inc. (Jersey City, NJ) and has equity interest in the same and in Tioma Therapeutics (St. Louis, MO).”

A correction has been made to **Conflict of Interest Statement**:

“JI serves as chair of the scientific advisory **board** of Radiation Control Technologies, Inc. (Jersey City, NJ) and has equity interest in the same and in Tioma Therapeutics (St. Louis, MO).”

The authors apologize for this error and state that this does not change the scientific conclusions of the article in any way.

## Conflict of interest statement

JI serves as chair of the scientific advisory board of Radiation Control Technologies, Inc. (Jersey City, NJ) and has equity interest in the same and in Tioma Therapeutics (St. Louis, MO). The other authors declare that the research was conducted in the absence of any commercial or financial relationships that could be construed as a potential conflict of interest.

