# Supplementary material for: Inhibition of VEGFR2 Activation and Its Downstream Signaling to ERK1/2 and Calcium by Thrombospondin-1 (TSP1): In silico Investigation
Source: Front Physiol. 2017 Feb 6;8:48. doi: 10.3389/fphys.2017.00048 (PMC5292565; doi:10.3389/fphys.2017.00048)
Supplement: Supplementary file 1 [file DataSheet1.PDF]

Supplementary Material

Inhibition of VEGFR2 activation and its downstream signaling to ERK1/2 and calcium by Thrombospondin-1 (TSP1): *In silico* investigation

Hojjat Bazzazi*, Jeffery S. Isenberg, and Aleksander S. Popel

*** Correspondence:** Corresponding Author: hbazzazi@jhmi.edu

1 Supplementary Figures and Tables

1.1 Supplementary Figures

1.2 Supplementary Tables

2 BioNetGen file

3 Supplementary References

1 Supplementary Figures and Tables

1.1 Supplementary Figures

Supplementary Figure 1.

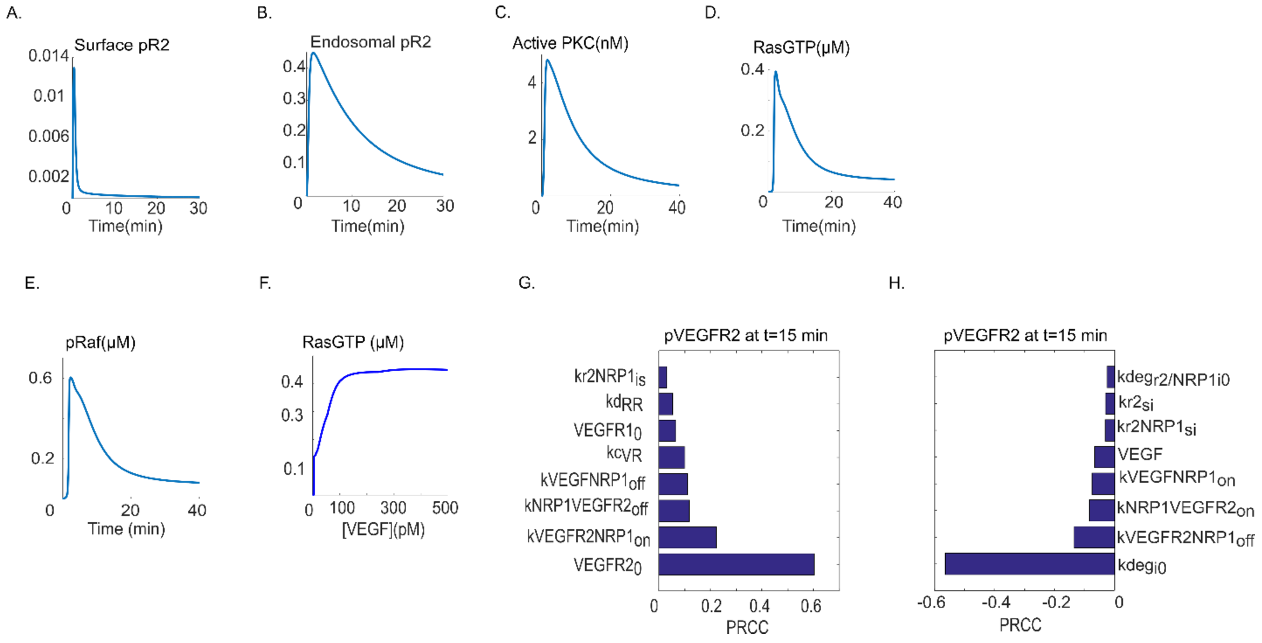

Figure S1. Predicted surface and endosomal pR2, PKC, RasGTP, and active Raf with sensitivity analysis of parameters important for VEGFR2 activation. A. Predicted surface pR2, B. Predicted endosomal pR2, (C-E) The predicted traces for active PKC, RasGTP, pRaf from the model, F. The dose response curve for RasGTP showing similar threshold behavior as pERK1/2, G. Positively correlated parameters from the partial rank correlation coefficient (PRCC) global sensitivity analysis, H. negatively correlated parameters.

Supplementary Figure 2.

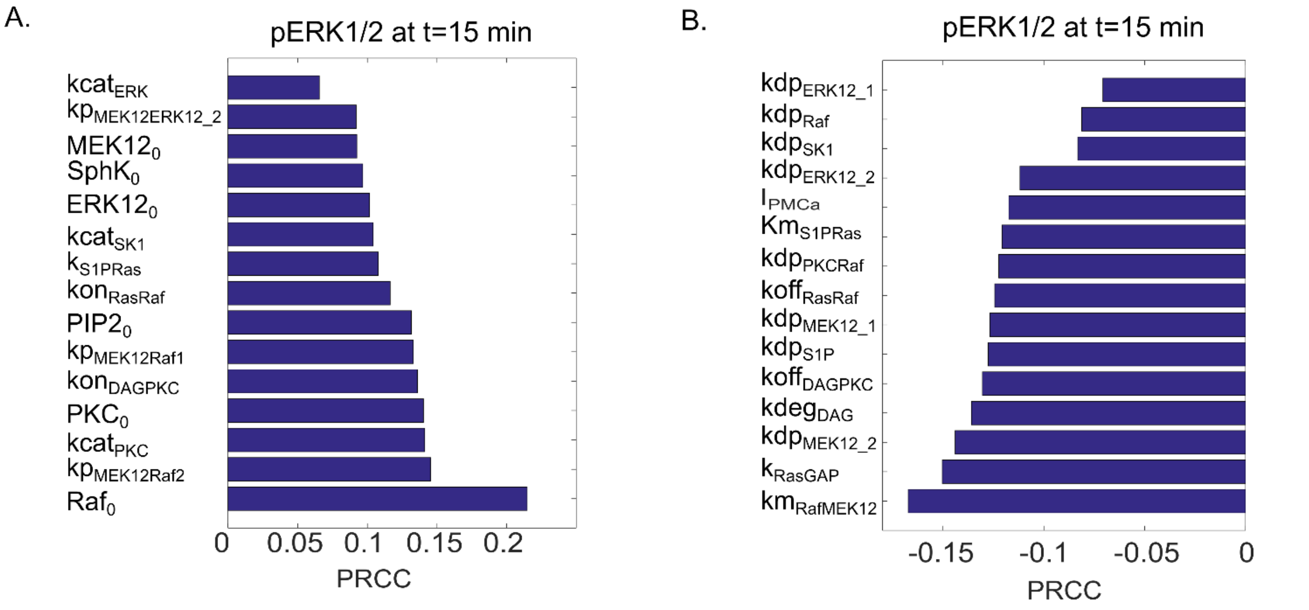

Figure S2. Global sensitivity analysis of the parameters important in ERK1/2 activation using PRCC algorithm. A. Ranking list of the positively correlated parameters with PRCC coefficients, B. Ranking list of the negatively correlated parameters along with the PRCC coefficients.

Supplementary Figure 3.

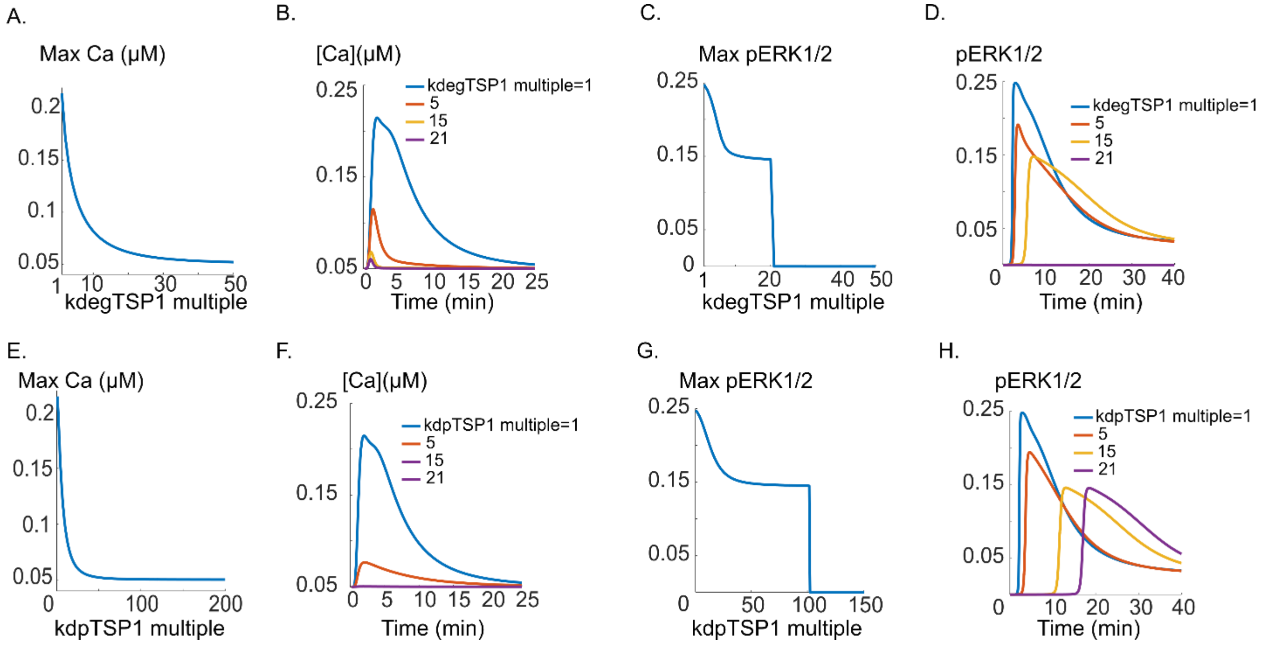

Figure S3. Simultaneous application of 2 nM TSP1 and 50 ng/ml VEGF to cells and the inhibition of VEGF signaling by mix of enhanced degradation and dephosphorylation mechanisms. (A-D) Assuming enhanced degradation of VEGFR2 as the sole mechanism for TSP1 effects/ I A. Maximum calcium signal decreases as a function of receptor degradation, B. Sample transients showing the inhibition of intracellular calcium, D. Maximum pERK1/2 is decreased as a function of receptor degradation showing a threshold behavior, E. pERK1/2 traces. (E-H) The case where increased VEGFR2 dephosphorylation by TSP1 is the dominant mechanism. E. The amplitude of the intracellular calcium decreases dephosphorylation rate is increased, F. Sample calcium traces for various levels of receptor dephosphorylation (fold-change relative to the control with no TSP1), G. pERK1/2 decreases as dephosphorylation rate is increased until a threshold value is reached (~90-fold increase relative to control value with no TSP1) is reached, beyond which there is no activation of ERK1/2, H. pERK1/2 versus time for various receptor dephosphorylation rates.

1.2 Supplementary Tables

Supplementary Table 1.

Table S1. Model parameters

**Name**	**Description**	**Value (units)**	**Reference**
Vol_{cyto}	Cytoplasmic volume	9.12×10^{-13} L	(Silva *et al.*, 2007)
Vol_{ext}	External volume	2×10^{-3} L	binding of ligands to surface receptors do not appreciably

			perturb the ligand concentration
Vol_{ER}	ER volume	3.35×10^{-13} L	(Silva *et al.*, 2007)
A_{cell}	Cell area	1400 μm^2	(Adams & Hill, 2004)
$VEGF165_0$	VEGF165A concentration	$1.19 \times 10^{-3} \mu M$ (50 ng/ml)	standard
$VEGFR2_0$	Total VEGFR2	4.29 # (number)/□μm^2 (6000 receptors per cell)	(Imoukhuede & Popel, 2012)
$VEGFR1_0$	Total VEGFR1	1.43 #/□μm^2 (2000 receptors per cell)	(Imoukhuede & Popel, 2012)
$NRP1_0$	Total NRP1	28.6 #/μm^2(40000 receptors)	(Imoukhuede & Popel, 2011)
$CD47free_0$	CD47 not pre-associated with VEGFR2. These receptors are in the background and bind TSP1, but do not participate in VEGFR2 signaling.	4.29 #/μm^2<br><br>(6000 receptors per cells)	No data available. Assumed to be similar to VEGFR2 levels.
kvr_{on}	The on kinetics of binding of VEGF to VEGFR2	4.4 $\mu M^{-1} s^{-1}$	(Mac Gabhann & Popel, 2007a)
kvr_{off}	Thee off kinetics of binding of VEGF to VEGFR2	2.6×10^{-2} s^{-1}	(Mac Gabhann & Popel, 2007a)
kc_{VR}	The on kinetics of binding of VEGF-VEGFRx species to VEGFRx receptors (x=1 or 2)	2.13×10^{-3} $\#^{-1} \mu m^2 s^{-1}$	Fit
kc_{RR}	Ligand-independent coupling of the receptors on kinetics	1.11 $\#^{-1} \mu m^2 s^{-1}$	Fit

kd_{RR}	Ligand-independent coupling of the receptors off kinetics	$0.78\ s^{-1}$	Fit
$kvr1_{on}$	The on kinetics of binding of VEGF to VEGFR1	$22\ \mu M^{-1} s^{-1}$	(Mac Gabhann & Popel, 2007a)
$kvr1_{off}$	The off kinetics of binding of VEGF to VEGFR1	$2.6\times10^{-2}\ s^{-1}$	(Mac Gabhann & Popel, 2007a)
kc_{RR}	The on kinetics of coupling of receptors within a hetero – or homo- dimer.	$2.50\ s^{-1}$	(Mac Gabhann & Popel, 2007a) and Fit
kc_{VR}	The on kinetics of binding of a receptor to VEGF within a hetero – or homo- dimer.	$2.05 s^{-1}$	(Mac Gabhann & Popel, 2007a) and Fit
kp_{Y1175}	Phosphorylation rate of Y1175	$4.239\times10^{3}\ s^{-1}$	Fit
kdp_{s}	De-phosphorylation rate of Y1175 at the membrane	$7.69\times10^{2}\ s^{-1}$	Fit
kdp_{i}	De-phosphorylation rate of Y1175 for internalized receptors	$5.44\ s^{-1}$	Fit
$kr2_{si}$	Rate of internalization of ligand-bound VEGFR2 without NRP1 in the complex	$6.1\times10^{-2}\ s^{-1}$	Fit
$kr2_{is}$	Endosome to membrane shuttling rate	$1.24\times10^{-3} s^{-1}$	Fit
$ksingleR2_{si}$	Internalization rate o VEGFR2 in the absence of ligand	$9.23\times10^{-4}\ s^{-1}$	Fit
$ksingleR2_{is}$	Endosome to membrane shuttling rate of VEGFR2 in the absence of ligand	$0.267\ s^{-1}$	Fit
$kdeg_{i0}$	Degradation rate of the internalized phosphorylated VEGFR2	$1.41\times10^{-3}\ s^{-1}$	Fit

$kdeg_{r2/NRP1i0}$	Degradation rate of the internalized phosphorylated VEGFR2 that are bound to at least one NRP1 co-receptor	$1.18 \times 10^{-2} s^{-1}$	Fit
$kdeg_{i0/notPhos}$	The degradation rate of un-phosphorylated VEGFR2 receptors that are not bound to NRP1 co-receptors	$9.37 \times 10^{-4}\ s^{-1}$	Fit
$kdeg_{r2/NRP1i0/notPhos}$	The degradation rate of unphosphorylated VEGFR2 receptors that are bound to at least one NRP1 co-receptor	$0.01\ s^{-1}$	Fit
$kr2NRP1_{si}$	Internalization rate of VEGFR2 with NRP1 in the complex	$0.404\ s^{-1}$	Fit
$kr2NRP1_{is}$	Endosome to membrane shuttling rate with NRP1in the complex	$0.756\ s^{-1}$	Fit
$kVEGFNRP1_{on}$	The on kinetics of binding of VEGF to NRP1	$3.2\ \mu M^{-1} s^{-1}$	(Mac Gabhann & Popel, 2007b)
$kVEGFNRP1_{off}$	The off kinetics of binding of VEGF to NRP1	$0.001 s^{-1}$	(Mac Gabhann & Popel, 2007b)
$kNRP1VEGFR2_{on}$	The on kinetics of VEGF.NRP1 binding to VEGFR2	$0.554\ \#^{-1} \mu m^2 s^{-1}$	Fit
$kNRP1VEGFR2_{off}$	The off kinetics of VEGF.NRP1 binding to VEGFR2	$4.891\ s^{-1}$	Fit
$kNRP1VEGFR1_{on}$	The on kinetics of NRP1 binding to VEGFR1	$1.31\ \#^{-1} \mu m^2 s^{-1}$	Fit
$kNRP1VEGFR1_{off}$	The off kinetics of NRP1 binding to VEGFR1	$0.1\ s^{-1}$	Fit
$kVEGFR2NRP1_{on}$	The on kinetics of binding of VEGF.VEGFR2 to NRP1	5.7×10^{-3} $\#^{-1} \mu m^2 s^{-1}$	Fit

$kVEGFR2NRP1_{off}$	The off kinetics of binding of VEGF.VEGFR2 to NRP1	5.05 s^{-1}	Fit
$kVEGFNRP1_{on}$	The on kinetics of binding of VEGF.VEGFR1 to NRP1	1.56×10^{-2} $\#^{-1}\mu m^2 s^{-1}$	Fit
$kVEGFNRP1_{off}$	The off kinetics of binding of VEGF.VEGFR1 to NRP1	5.36s^{-1}	Fit
$kPIP2_{gen}$	Rate of PIP2 generation	$4.8\times10^{-5}s^{-1}$	(Zhang *et al.*, 2014)
$kSph_{gen}$	Rate of Sphingosine generation	$4.8\times10^{-5}s^{-1}$	Same as PIP2
$kp_{PLC\gamma}$	Phosphorylation rate of PLCγ	0.1 $(\#/\mu m^2)^{-1}s^{-1}$	Fit
$Km_{PLC\gamma/R2}$	Michaelis-Menten type constant for the activation of PLCγ by phospho-VEGFR2	8 μM	FIt
$kdp_{PLC\gamma}$	De-phosphorylation rate of PLCγ	0.1 s^{-1}	Fit
$km_{PIP2PLC\gamma}$	Michaelis-Menten type parameter for PLCγ phosphorylation	0.194 μM	Fit
n_{DAG}	The Hill coefficient for the generation of IP3 and DAG by PLCγ	2.495	Fit
$kcat_{PLC\gamma.DAG}$	The catalytic rate of PLCγ	0.1 s^{-1}	Fit
$k_{deg,IP3}$	Degradation rate of IP3	9.22×10^{-2}	Fit
$k_{deg,DAG}$	Degradation rate of DAG	0.109 s^{-1}	Fit
I_{ip3R}	Amplitude of the IP3R current	3.62×10^{4} μMs^{-1}	Fit
$K_{m,IP3R}$	IP3 concentration for half-maximal activation of IP3R	1.6μM	(Wiesner *et al.*, 1996; Carter & Ogden, 1997)

I_{PMCA}	Maximum plasma membrane calcium pump current	5.98 μMs^{-1}	Fit
$K_{m,PMCA}$	Calcium concentration fohalf maximal plasma membrane pump activity	0.26 γM	{Silva, 2007 #387}
I_{SERCA}	Maximum ER calcium pump current	4.77 μMs^{-1}	Fit
$Kleak_{ER}$	ER Calcium leak parameter	7.48×10^{-8} $\mu M^{-1}s^{-1}$	Fit
Km_{SERCA}	Calcium concentration for half maximal activity of the ER calcium pump	0.15 μM	(Wiesner *et al.*, 1996)
$K_{i,Ca}$	Ca inhibition of IP3R	1 μM	(Wiesner *et al.*, 1996)
$K_{a,Ca}$	Ca activation of IP3R	0.1 μM	(De Young & Keizer, 1992)
$k_{B,on}$	The on kinetics of binding of calcium to cytoplasmic calcium buffers	100 $\mu M^{-1}s^{-1}$	(Wiesner *et al.*, 1996)
$k_{B,off}$	The off kinetics of binding of calcium to cytoplasmic calcium buffers	300 s^{-1}	(Wiesner *et al.*, 1996)
$kon_{Ca/NCaM1}$	The on rate for the binding of calcium to the first EF-hand domain on the N-lobe of calmodulin	25 $\mu M^{-1}s^{-1}$	(Pepke *et al.*, 2010)
$koff_{Ca/NCaM1}$	The off rate for the binding of calcium to the first EF-hand domain on the N-lobe of calmodulin	1000 s^{-1}	(Pepke *et al.*, 2010)
$kon_{Ca/NCaM2}$	The on rate for the binding of calcium to the second EF-hand domain on the N-lobe of	50 $\mu M^{-1}s^{-1}$	(Pepke *et al.*, 2010)

	calmodulin		
$koff_{Ca/NCaM2}$	The off rate for the binding of calcium to the second EF-hand domain on the N-lobe of calmodulin	500 s^{-1}	(Pepke *et al.*, 2010)
$kon_{Ca/CCaM1}$	The on rate for the binding of calcium to the first EF-hand domain on the C-lobe of CaM	1.2 $\mu M^{-1}s^{-1}$	(Pepke *et al.*, 2010)
$koff_{Ca/CCaM1}$	The off rate for the binding of calcium to the first EF-hand domain on the C-lobe of CaM	10 s^{-1}	(Pepke *et al.*, 2010)
$kon_{Ca/CCaM2}$	The on rate for the binding of calcium to the second EF-hand domain on the C-lobe of CaM	5 $\mu M^{-1}s^{-1}$	(Pepke *et al.*, 2010)
$koff_{Ca/CCaM2}$	The off rate for the binding of calcium to the second EF-hand domain on the C-lobe of CaM	8.5 s^{-1}	(Pepke *et al.*, 2010)
$CSQN_{total}$	Total concentration of calsequesterin (ER calcium buffer)	15000 μM	(Winslow *et al.*, 2000)
Kd_{CSQN}	Dissociation constant for the binding of calcium to CSQN	800 μM	(Winslow *et al.*, 2000)
$kon_{Ca/PKC}$	The on rate for the binding of calcium to PKC	0.3 $\mu M^{-1}s^{-1}$	(Bhalla *et al.*, 2002)
$koff_{Ca/PKC}$	The off rate for the binding of calcium to PKC	0.01 s^{-1}	(Bhalla *et al.*, 2002)
$kon_{DAG/PKC}$	The on rate of DAG binding to PKC	0.030 $\mu M^{-1}s^{-1}$	Fit
$koff_{DAG/PKC}$	The off rate of DAG binding to PKC	0.124 s^{-1}	Fit
$kon1_{Ca/CIB}$	The on rate of calcium binding to the first EF-hand domain of CIB1	0.0526 $\mu M^{-1}s^{-1}$	Fit (Kd=1.9 μM from (Yamniuk *et al.*, 2004))

$koff1_{Ca/CIB1}$	The off rate of calcium binding to the first EF-hand domain of CIB1	0.1 s^{-1}	Fit
$kon2_{Ca/CIB1}$	The on rate of calcium binding to the second EF-hand domain of CIB1	0.185 $\mu M^{-1} s^{-1}$	Fit (Kd=0.54 μM from (Yamniuk *et al.*, 2004))
$koff2_{Ca/CIB1}$	The off rate of calcium binding to the second EF-hand domain of CIB1	0.1 s^{-1}	Fit
$kon_{CIB1/SphK1}$	The on rate for the binding of CIB1 to SphK1	17.603 $\mu M^{-1} s^{-1}$	Fit
$koff_{CIB1/SphK1}$	The off rate for the binding of CIB1 to SphK1	4.403 s^{-1}	Fit
$kcat_{SK1}$	The catalytic rate of SphK1	37.238 s^{-1}	Fit
$Km_{SK1/Sph}$	The Michaelis-Menten type parameter for SphK1 enzymatic activity	0.0294 μM	Fit
kt_{SK1}	Translocation rate of CIB1-bound SphK1 from cytoplasm to the membrane	1 s^{-1}	Fit
$koff_{SK1}$	The off rate of calcium-free CIB1/SphK1 from the membrane	0.104 s^{-1}	Fit
kdp_{SK1}	Dephosphorylation rate of SphK1	0.0218 s^{-1}	Fit
k_{RasGAP}	The rate of RasGTP hydrolysis by RasGAP	2.941 s^{-1}	Fit
$kon_{Ras/Raf}$	The on rate for the binding of activated Ras to Raf	13.102 $\mu M^{-1} s^{-1}$	Fit
$koff_{Ras/Raf}$	The off rate for the binding of activated Ras to Raf	0.152 s^{-1}	Fit

kp_{Raf}	The rate of autophosphorylation of the activating tyrosine residues on Raf	1.676 s^{-1}	Fit
kdp_{Raf}	The rate of dephosphorylation of Raf for the tyrosine residues	0.895 s^{-1}	Fit
$kdp_{PKC/Raf}$	The rate of dephosphorylation of the PKC-phosphorylated serine residue on Raf	0.720 s^{-1}	Fit
$kp_{MEK12/Raf1}$	The rate of phosphorylation of the first serine residue on MEK1/2 by activated Raf	1.802 s^{-1}	Fit
$kp_{MEK12/Raf2}$	The rate of phosphorylation of the second serine residue on MEK1/2 by activated Raf	1.205 s^{-1}	Fit
$Km_{MEK12/Raf}$	The Michaelis-Menten type parameter for the phosophorylation of MEK1/2 by active Raf	0.807 μM	Fit
kdp_{MEK12_1}	Rate of dephosphorylation of the first serine on MEK1/2	0.112 s^{-1}	Fit
kdp_{MEK12_2}	Rate of dephosphorylation of the second serine on MEK1/2	0.140 s^{-1}	Fit
$kp_{MEK12/ERK12\_1}$	Phosphorylation rate of ERK1 by active MEK1/2	12.149 s^{-1}	Fit
$kp_{MEK12/ERK12\_2}$	Phosphorylation rate of ERK2 by active MEK1/2	0.516 s^{-1}	Fit
kdp_{ERK12_1}	Dephosphorylate rate of ERK1	6.06 s^{-1}	Fit
kdp_{ERK12_2}	Dephosphorylation rate of ERK2	1.053 s^{-1}	Fit
$kcat_{ERK}$	Catalytic rate of active ERK1/2	7.883 s^{-1}	Fit
$Km_{ERK/SK1}$	The Michaelis-Menten type constant for the phosphorylation of	1.198 μM	Fit

	SphK1 by active ERK1/2		
$k_{S1P/Ras}$	Rate constant for the activation of Ras by S1P	1.556 μMs^{-1}	Fit
$Km_{S1P/Ras}$	Concentration of S1P for half-maximal rate of Ras activation	5.899 μM	Fit
kdp_{S1P}	Rate of S1P dephosphorylation	1.188	Fit
$kcat_{PKC}$	The catalytic rate of PKC	10.208 s^{-1}	Fit
$Km_{PKC/Raf}$	The Michaelis-Menten type constant for the phosphorylation of Raf by PKC	0.314 μM	Fit
I_{CRAC}	The amplitude of the CRAC channel current	1.74×10^{4} $\mu M^{-1}s^{-1}$	Fit
K_{CRAC}	ER concentration resulting in half-maximal CRAC current at steady-state	169 μM	(Luik *et al.*, 2008)
n_{CRAC}	Hill number for the steady-state CRAC channel activation	4.2	(Luik *et al.*, 2008)
I_{stim}	The time constant for the activation of the CRAC current	4 s	Fit and (Schmeitz *et al.*, 2013)
$ktoff_{SK1}$	The off rate of CIB1/SphK1 binding to the plasma membrane	6.67×10^{-4} s^{-1}	Fit
$Kr2TSP1_{si}$	The rate of internalization of TSP1/CD47/VEGF/VEGFR2 complex	6.1×10^{-2} s^{-1} (varied)	Initially the same as $kr2_{si}$
$Kr2NRP1TSP1_{si}$	The rate of internalization of TSP1/CD47/VEGF/VEGFR2/NRP1 complex	0.404 s^{-1}	varied

$kdegTSP1_{i0}$	Degradation rate of the internalized phosphorylated VEGFR2 with TSP1 bound to CD47 in the complex. Without NRP1 in the complex.	1.41×10^{-3} s^{-1}	varied
$kdegTSP1_{i0/notPhos}$	Degradation rate of the internalized un-phosphorylated VEGFR2 with TSP1 bound to CD47 without NRP1 in the complex	9.37×10^{-4} s^{-1}	varied
$kdegTSP1NRP1_{i0}$	Degradation rate of phosphorylated VEGF/VEGFR2/NRP1/CD47 complex with TSP1	$1.18\times10^{-2}s^{-1}$	varied
$kdegTSP1NRP1_{i0/notPhos}$	Degradation of un-phosphorylated VEGF/VEGFR2/NRP1/CD47 complex with TSP1 bound to CD47	0.01 s^{-1}	varied
$kCD47TSP1_{off}$	The off kinetics for the binding of CD47 to TSP1	0.001 s^{-1}	varied
$kD_{CD47/TSP1}$	The dissociation constant for the binding of CD47 and TSP1	10 pM	(Isenberg *et al.*, 2009)
$kcd47free_{on}$	The on rate for the irreversible binding between single CD47 and VEGFR2	0.001 s^{-1}	Assumed

Supplementary Table 2.

Table S2. Initial values for the seed species in the model

**Name**	**Value (units)**
PLC□□	0.2 μM
$CaER_0$	2000 μM
$Calcium_0$	0.050 μM

CaF_0	118.03 μM
$CaFbound_0$	1.97 μM
PKC_0	0.1 μM
$CIB1_0$	0.5 μM
$SphK1_0$	0.1 μM
Sph_0	10 μM
$S1P_0$	0 μM
$RasGDP_0$	0 μM
$RasGTP_0$	0 μM
Raf_0	0.355 μM
$MEK1/2_0$	0.289 μM
$ERK1/2_0$	0.382 μM
$Istim_0$	0.542 μM/s

2 BioNetGen file

```
begin model
begin parameters
Volcyto 9.12E-13
Volext  0.0005
VolER 3.35E-13
fextmolar 1.205E+15
fcmolar 549000
cellarea  1400
```

VEGF165a_0 0.001190476

VEGFR2_0 4.29E+00

VEGFR1_0 1.428571429

NRP1_0 2.86E+01

Calcium_0 0.05010232

CaM_0 1

kvron 4.4

kvroff 2.60E-02

kcVR 0.002126712

kcRR 1.109768189

kdRR 0.784915318

kvr1on 22

kvr1off 0.026

kdeltaRR 2.503952539

kdeltaVR 2.052324738

kpY1175 4239

kdps 769.0416257

kdpi 5.438985303

kdpTSP1s 769.0416257

kdpTSP1i 769.0416257

kr2si 0.060670655

kr2is 0.001242368

kr2NRP1si 0.404165983

kr2NRP1is 0.755627395

kVEGFNRP1on 3.2

kVEGFNRP1off 0.001

kNRP1VEGFR2on 0.553575486

kNRP1VEGFR2off 4.89126933

kNRP1VEGFR1on 1.314223544

kNRP1VEGFR1off 840.6509211

kVEGFR2NRP1on 0.005707355

kVEGFR2NRP1off 5.051782424

kVEGFR1NRP1on 0.015561472

kVEGFR1NRP1off 5.355945178

PIP2_0 10

kPIP2gen 0.000048

kSphgen 0.000048

kpPLCgamma 0.1

kdpPLCgamma 0.1

PLCgamma_0 0.2

kmPIP2PLCgamma 0.193585826

nDAG 2.495436003

kcatPLCgammaDAG 0.10046696

kdeg_ip3 0.0921875

kdeg_DAG 0.108898602

DAG_0 0

IP3_0 0

CaER_0 2.00E+03

Iip3Ramp 3.62E+04

KmIP3R 1.6

I_PMCAbar 5.978039695

KmPMCA 0.26

Caext 2000

KpmLeak 0

I_ERCA 4.76870897

KleakER 7.48E-08

KmERCA 0.15

KiCa 1

KaCa 0.1

KBon 100

KBoff 300

CaF_0 118.0327869

CaFbound_0 1.967213115

konCaNCaM1 25

koffCaNCaM1 1000

konCaNCaM2 50

koffCaNCaM2 500

konCaCCaM1 1.2

koffCaCCaM1 10

konCaCCaM2 5

koffCaCCaM2 8.5

CSQN_total 15000

KCSQN 800

PKC_0 0.1

konCaPKC 0.3

koffCaPKC 0.01

konDAGPKC 0.029957319

koffDAGPKC 0.124096212

kon1CaCIB1 0.052631579

koff1CaCIB1 0.1

kon2CaCIB1 0.185185185

koff2CaCIB1 0.1

CIB1_0 0.5

konCIB1SphK1 17.6028519

koffCIB1SphK1 4.403956392

SphK_0 0.1

Sph_0 10

S1P_0 0

KmSK1Sph 0.029430478

kcatSK1Sph 37.23820691

ktSK1 1

koffSK1 1.04E-01

kdpSK1 0.02182773

RasGTP_0 0

RasGDP_0 0.1

kRasGAP 2.941097467

kdegi0 1.41E-03

kdegTSP1i0 1.41E-03

kdegTSP1i0noUB 9.37E-04

kdegTSP1NRP1i0 1.18E-02

kdegTSP1NRP1i0noUB 1.00E-02

Raf_0 0.355471965

MEK12_0 0.288919159

ERK12_0 0.382329627

konRasRaf 13.10183719

koffRasRaf 0.151878139

kpRaf 1.67558904

kdpRaf 0.894826206

kdpPKCRaf 0.720296112

KmMEK12Raf 0.807388937

kpMEK12Raf1 1.801786102

kdpMEK12_1 0.111827134

kdpMEK12_2 0.139705453

kmMEKERK12 0.25546079

kpMEK12ERK12_1 12.14930177

kdpERK12_1 6.06E+00

kdpERK12_2 1.053392443

kcatERK 7.882741692

kmERKSK1 1.198236617

kS1PRas 1.556423632

KmS1PRas 5.899306591

kdpS1P 1.188017664

kcatPKC 10.20798409

kmPKCRaf 0.313875333

ksingleR2syn 4.05E-08

ksingleR2si 9.23E-04

ksingleR2TSP1si 9.23E-04

ICrac 1.74E+04

Kcrac 169

kpMEK12Raf2 1.204676328

kpMEK12ERK12_2 0.516270813

kdegr2NRP1i0 1.18E-02

tau_stim 4

kmPLCgammaR2 8

Istim0 0.542138034

ncrac 4.2

ktoffSK1 6.97E-04

kdegi0noUB 9.37E-04

kdegr2NRP1i0noUB 1.00E-02

kr2isnoUB 0.267258037

kr2TSP1si 0.060670655

kr2NRP1TSP1si 0.404165983

koffCD47TSP1 0.001

kDCD47TSP1 1e-5

kcd47free_on 1

TSP1_0 2e-3

CD47free_0 4.29

end parameters

begin molecule types

vegf(r,r,nrp1bd,c~s~i) # vegf165a

vegfr2(l1,Y1175~Y~pY,CD47bd,dimer,c~s~i)

vegfr1(l2,dimer,nrp1bd,Yr1~Y~pY,c~s)

NRP1(vegfabd,c~s~i)

PI(PIsite~3P~4P)

PLCgamma(R2binding,Yplc~Y~pY)

DAG(pkcbd)

IP3_cyto(ip3rbd)

Calcium_cyto(bd)

Trash()

CaER(bd)

CaF(cabd)

CaM(NCaM1,NCaM2,CCaM1,CCaM2,CaMtargetbd)

PKC(CalciumBD,DAGBD)

I()

CIB1(EF1,EF2,sk1bd,location~cytosol~membrane)

SphK(CIB1bd,Serk~S~pS,Spkc~S~pS)

Sph(skbd)

S1P(bd)

RasGDP(rafbd)

RasGTP(rafbd)

Raf(mekbd,rasbd,Y1Y2~Y~pY,Spkc~S~pS,S259~S~pS)

MEK12(bd,S1~S~pS,S2~S~pS)

ERK1(MEK12bd,S1~S~pS,dimer)

ERK2(MEK12bd,S2~S~pS,dimer)

Istim()

CD47SIRPa(VEGFR2bd,TSP1bd,c~s~i)

TSP1(CD47bd)

end molecule types

begin seed species

vegf(r,r,nrp1bd,c~s) VEGF165a_0

vegfr1(l2,dimer,nrp1bd,Yr1~Y,c~s) VEGFR1_0

vegfr2(l1,Y1175~Y,CD47bd,dimer,c~s) VEGFR2_0

NRP1(vegfabd,c~s) NRP1_0

PI(PIsite~3P) PIP2_0

PLCgamma(R2binding,Yplc~Y) PLCgamma_0

CaER(bd) CaER_0

```
Calcium_cyto(bd)         Calcium_0

CaF(cabd)         CaF_0

Calcium_cyto(bd!1).CaF(cabd!1)  CaFbound_0

    CaM(NCaM1,NCaM2,CCaM1,CCaM2,CaMtargetbd) CaM_0

 PKC(CalciumBD,DAGBD)       PKC_0

 I()                1

 CIB1(EF1,EF2,sk1bd,location~cytosol)        CIB1_0

 SphK(CIB1bd,Serk~S,Spkc~S)    SphK_0

 Sph(skbd)     Sph_0

 S1P(bd)      S1P_0

 RasGDP(rafbd)    RasGDP_0

 RasGTP(rafbd)    RasGTP_0

 Raf(mekbd,rasbd,Y1Y2~Y,Spkc~S,S259~S)  Raf_0

 MEK12(bd,S1~S,S2~S)  MEK12_0

 ERK1(MEK12bd,S1~S,dimer)    ERK12_0

 ERK2(MEK12bd,S2~S,dimer)   ERK12_0

 Istim()      Istim0

TSP1(CD47bd)  TSP1_0

CD47SIRPa(VEGFR2bd,TSP1bd,c~s)    CD47free_0

end seed species


begin observables

Molecules   VEGFR2tots  vegfr2(c~s)

 Molecules   VEGFR2toti  vegfr2(c~i)

Molecules  VEGFR2total vegfr2()

  Molecules    vegfr2Y1175ps  vegfr2(Y1175~pY!?,c~s)

  Molecules    vegfr2Y1175pi  vegfr2(Y1175~pY!?,c~i)

  Molecules   vegffrees  vegf(r,r,nrp1bd,c~s)

  Molecules    freeDAGs      DAG(pkcbd!?)

  Molecules     activePLCgamma  PLCgamma(Yplc~pY!?)

  Molecules      boundPLCgamma   PLCgamma(R2binding!+,Yplc~Y)

  Molecules     activePLCgammafree  PLCgamma(R2binding,Yplc~pY)
```

Molecules activePLCgammabound PLCgamma(R2binding!+,Yplc~pY)

Molecules PIP2 PI(PIsite~3P)

Molecules PIP3 PI(PIsite~4P)

Molecules freeip3cyto IP3_cyto(ip3rbd)

Molecules Cac Calcium_cyto(bd)

Molecules Caer CaER(bd)

Molecules CaBuf_fer CaF(cabd!+)

Molecules activePKCs PKC(CalciumBD!+,DAGBD!1).DAG(pkcbd!1)

Molecules activePKCtot PKC(CalciumBD!+,DAGBD!+)

Molecules SphKpkc SphK(Serk~pS!?)

Molecules S1phosphate S1P(bd)

Molecules singleNRP1vefr1s vegf(r!1,r,nrp1bd!+,c~s).vegfr1(l2!1,c~s)

Molecules NRP1frees NRP1(vegfabd,c~s)

Molecules NRP1freei NRP1(vegfabd,c~i)

Molecules NRP1bounds NRP1(vegfabd!+,c~s)

Molecules NRP1boundi NRP1(vegfabd!+,c~i)

Molecules vr2s vegfr2(l1,c~s)

Molecules vr2i vegfr2(l1,c~i)

Molecules vr2dimers vegfr2(l1,dimer!1,c~s).vegfr2(l1,dimer!1,c~s)

Molecules vr2dimeri vegfr2(l1,dimer!1,c~i).vegfr2(l1,dimer!1,c~i)

Molecules vr1s vegfr1(l2,nrp1bd,c~s)

Molecules vr1dimers vegfr1(l2,dimer!1,nrp1bd,c~s).vegfr1(l2,dimer!1,nrp1bd,c~s)

Molecules vegfr1NRP1frees vegfr1(l2,nrp1bd!+,c~s)

Molecules vegfr1NRP1bs vegfr1(l2!1,nrp1bd!+,c~s).vegf(r!1,r,nrp1bd,c~s)

Molecules vegfr1NRP1b2s vegfr1(l2!+,nrp1bd!+,c~s)

Molecules vr12dimers vegfr1(l2,dimer!1,nrp1bd,c~s).vegfr2(l1,dimer!1,c~s)

Molecules vr12dimertotals vegfr1(l2,dimer!1,c~s).vegfr2(l1,dimer!1,c~s)

Molecules vr1totals vegfr1(l2,dimer,c~s)

Molecules vr1dimertotals vegfr1(l2,dimer!1,c~s).vegfr1(l2,dimer!1,c~s)

Molecules vr2Y1175s vegfr2(Y1175~pY,c~s)

Molecules rasgdpfree RasGDP(rafbd!?)

Molecules rasgtpfree RasGTP(rafbd!?)

Molecules activeRafbyrastot Raf(Y1Y2~pY!?)

Molecules phosphoMEK12tot MEK12(S1~pS!?,S2~pS!?)

Molecules phosphoERK1tot ERK1(S1~pS!?)

Molecules phosphoERK2tot ERK2(S2~pS!?)

Molecules dimerERKtotal ERK1(dimer!+)

Molecules vegfr1pY1s vegfr1(Yr1~pY,c~s)

Molecules SphK1 SphK(CIB1bd!+,Serk~S).CIB1(sk1bd!1,location~membrane)

Molecules SphK1mempS SphK(CIB1bd!+,Serk~pS).CIB1(sk1bd!1,location~membrane)

Molecules SphK1cytosol SphK(CIB1bd!+,Serk~S).CIB1(sk1bd!1,location~cytosol)

Molecules activeSphK1 SphK(Serk~pS!?)

Molecules freeSK1 SphK(CIB1bd,Serk~S,Spkc~S)

Molecules freeSK1mem SphK(CIB1bd!1,Serk~S).CIB1(sk1bd!1,location~membrane)

Molecules freecib1 CIB1(EF1,EF2,sk1bd)

Molecules calciumcib1 CIB1(EF1!+,EF2!+,sk1bd)

Molecules sk1bcib1 CIB1(EF1!+,EF2!+,sk1bd!+)

Molecules nrp1s NRP1(vegfabd,c~s)

Molecules vegfnrp1s vegf(r,r,nrp1bd!1,c~s).NRP1(vegfabd!1,c~s)

Molecules vegfr1s vegf(r!1,r,nrp1bd,c~s).vegfr1(l2!1,c~s)

Molecules vegfr2s vegf(r!1,r,nrp1bd,c~s).vegfr2(l1!1,dimer,c~s)

Species plcgammar2s PLCgamma(R2binding!1,Yplc~Y).vegfr2(Y1175~pY!1,c~s)

Species plcgammar2i PLCgamma(R2binding!1,Yplc~Y).vegfr2(Y1175~pY!1,c~i)

Species activeplcgammar2s PLCgamma(R2binding!1,Yplc~pY).vegfr2(Y1175~pY!1,c~s)

Species activeplcgammar2i PLCgamma(R2binding!1,Yplc~pY).vegfr2(Y1175~pY!1,c~i)

Species activeNCaM CaM(NCaM1!+,NCaM2!+)

Species activeCCaM CaM(CCaM1!+,CCaM2!+)

Species activeCaM CaM(NCaM1!+,NCaM2!+,CCaM1!+,CCaM2!+)

Molecules freepip2 PI(PIsite~3P)

Species vr2pY1175plcgammas PLCgamma(R2binding!1,Yplc~Y).vegfr2(Y1175~pY!1,c~s)

Species vr2pY1175plcgammai PLCgamma(R2binding!1,Yplc~Y).vegfr2(Y1175~pY!1,c~i)

Molecules freesphingosin Sph(skbd)

Molecules freeraf Raf(Spkc~S)

Molecules activeRafPKC   Raf(Spkc~pS!?)

Molecules activeRafPKCERK1 Raf(Spkc~pS!?,Y1Y2~pY!?)

Molecules activeRafPKCERK2 Raf(Spkc~pS!?,Y1Y2~Y!?)

Molecules activeRafPKCERK3 Raf(Spkc~S!?,Y1Y2~pY!?)

Molecules rafY1Y2pY Raf(Y1Y2~pY,Spkc~S)

Molecules rafY1Y2pYpS   Raf(Y1Y2~pY,Spkc~pS)

Molecules rafpS   Raf(Y1Y2~Y,Spkc~pS)

Molecules erk1s ERK1(S1~S)

Molecules mek12s   MEK12(S1~S,S2~S)

Molecules R2singlei vegfr2(l1,Y1175~Y,dimer,c~i)

Molecules pERK1s ERK1(S1~pS)

Molecules mek12ps   MEK12(S1~pS,S2~S)

Molecules pERK2s ERK2(S2~pS)

Molecules mek12ps1 MEK12(S1~pS)

Molecules mek12ps2 MEK12(S2~pS)

Molecules erk1ps ERK1(S1~pS)

Molecules erk2ps ERK2(S2~pS)

Molecules vr2r2py1175s vegfr2(Y1175~pY,c~s)

Molecules vr2r2py1175i vegfr2(Y1175~pY,c~i)

Species vr2py1175s   vegf(r!1,r,c~s).vegfr2(l1!1,Y1175~pY,c~s)

Species vr2py1175i   vegf(r!1,r,c~i).vegfr2(l1!1,Y1175~pY,c~i)

Molecules r2singlepy1175s vegfr2(l1,Y1175~pY,c~s)

Molecules r2singlepy1175i vegfr2(l1,Y1175~pY,c~i)

Molecules vr1r2pY1165s vegf(r!1,r!2,c~s).vegfr1(l2!1,c~s).vegfr2(l1!2,Y1175~pY,c~s)

Molecules plcfreepY PLCgamma(R2binding,Yplc~pY)

Molecules plcboundpY PLCgamma(R2binding!+,Yplc~pY)

Molecules pSK1 SphK(Serk~pS)

Molecules prafpkcs Raf(Spkc~pS)

Molecules frees1p S1P(bd)

Molecules py1y2rafs Raf(Y1Y2~pY)

Molecules nrp1i NRP1(vegfabd,c~i)

Molecules vegfnrp1i vegf(r,r,nrp1bd!1,c~i).NRP1(vegfabd!1,c~i)

Species vegfr2i vegf(r!1,r,nrp1bd,c~i).vegfr2(l1!1,c~i)

Molecules totalnrp1 NRP1(vegfabd)

Molecules pplcgamma PLCgamma(Yplc~pY)

Molecules phosphoERKpS1 ERK1(S1~pS!?)

Molecules phosphoERKpS2 ERK2(S2~pS!?)

Molecules phosphoMEKpS1 MEK12(S1~pS!?,S2~S)

Molecules phosphoMEKpS2 MEK12(S1~S,S2~pS!?)

Molecules freeSphK1 SphK(CIB1bd,Serk~S)

Molecules rafpkc Raf(Spkc~pS)

Molecules mek12s1 MEK12(S1~S)

Molecules mek12s2 MEK12(S2~S)

Molecules erk12s1 ERK1(S1~S)

Molecules erk12s2 ERK2(S2~S)

Molecules yplcgamma PLCgamma(Yplc~Y)

Molecules gtpfreeras RasGTP(rafbd)

Molecules vegfr2UB vegfr2()

Molecules sk1pkcpS SphK(Spkc~S)

Molecules freeskmem CIB1(sk1bd!1,location~membrane).SphK(CIB1bd!1,Spkc~S)

Molecules pSK1pkc SphK(Spkc~pS)

Molecules vr2r2py1054s vegfr2(Y1175~pY,c~s)

Molecules vr2r2py1054i vegfr2(Y1175~pY,c~i)

Molecules bvegfr2 vegf(r!1,nrp1bd,c~s).vegfr2(l1!1,c~s)

Molecules bnrp1 vegf(nrp1bd!1,c~s).NRP1(vegfabd!1,c~s)

Molecules bvegfr1 vegf(r!1,nrp1bd,c~s).vegfr1(l2!1,c~s)

Molecules bvegfr1dimer vegf(r!1,r!2,c~s).vegfr1(l2!1,c~s).vegfr1(l2!2,c~s)

Molecules bvegfr2_2 vegf(r!1,c~s).vegfr2(l1!1,c~s)

Molecules bvegfr1_2 vegf(r!1,c~s).vegfr1(l2!1,c~s)

Molecules vegfbound_1 vegf(r!+,nrp1bd,c~s)

Species vegfbound_2 vegf(r,nrp1bd!+,c~s)

Molecules vegfbound_3 vegf(r!+,c~s)

Species vegfbound_4 vegf(r,r,nrp1bd!1,c~s).NRP1(vegfabd!1,c~s)

```
Molecules Iopenstim    Istim()

Molecules cd47s  CD47SIRPa(TSP1bd,c~s)

Molecules cd47tsp1s TSP1(CD47bd!1).CD47SIRPa(TSP1bd!1,c~s)

Molecules cd47tsp1i TSP1(CD47bd!1).CD47SIRPa(TSP1bd!1,c~i)

Molecules CD47SIRPbs CD47SIRPa(TSP1bd!+,c~s)

Molecules CD47SIRPbi CD47SIRPa(TSP1bd!+,c~i)

Molecules  vegfr1r2s vegf(r!1,nrp1bd).vegfr1(l2!1,c~s)

Molecules freeVEGFR2s vegfr2(CD47bd,c~s)

Molecules freeVEGFR2i vegfr2(CD47bd,c~i)

Molecules freeVEGFR2tot vegfr2(CD47bd)

Molecules tsp1frees TSP1(CD47bd)

end observables


begin reaction rules



vegfr2(l1,dimer,c~s) + vegfr2(l1,dimer,c~s) <->  \

vegfr2(l1,dimer!1,c~s).vegfr2(l1,dimer!1,c~s) kcRR,kdRR

vegfr2(l1,dimer,c~s) + vegfr1(l2,dimer,c~s) <-> \

vegfr2(l1,dimer!1,c~s).vegfr1(l2,dimer!1,c~s) kcRR,kdRR

vegfr1(l2,dimer,c~s) + vegfr1(l2,dimer,c~s) <-> \

vegfr1(l2,dimer!1,c~s).vegfr1(l2,dimer!1,c~s) kcRR,kdRR


vegf(r!1,r!2,c~s).vegfr2(l1!1,dimer,c~s).vegfr2(l1!2,dimer,c~s) <-> \

vegf(r!1,r!2,c~s).vegfr2(l1!1,dimer!3,c~s).vegfr2(l1!2,dimer!3,c~s)   kdeltaRR,kdRR

vegf(r!1,r!2,c~s).vegfr2(l1!1,dimer,c~s).vegfr1(l2!2,dimer,c~s) <-> \

vegf(r!1,r!2,c~s).vegfr2(l1!1,dimer!3,c~s).vegfr1(l2!2,dimer!3,c~s)   kdeltaRR,kdRR

vegf(r!1,r!2,c~s).vegfr1(l2!1,dimer,c~s).vegfr1(l2!2,dimer,c~s) <-> \

vegf(r!1,r!2,c~s).vegfr1(l2!1,dimer!3,c~s).vegfr1(l2!2,dimer!3,c~s)   kdeltaRR,kdRR


# VEGFR1 binding to NRP1
```

```
vegfr1(nrp1bd,c~s) + NRP1(vegfabd,c~s) <-> vegfr1(nrp1bd!1,c~s).NRP1(vegfabd!1,c~s) kNRP1VEGFR1on,kNRP1VEGFR1off


#Binding of VEGF165a to NRP1

I() -> I() + vegf(r,r,nrp1bd,c~s)  -
(kVEGFNRP1on*cellarea/fextmolar)*vegffrees*nrp1s+(kVEGFNRP1off*cellarea/fextmolar)*vegfnrp1s

vegf(r,r,nrp1bd,c~s) + NRP1(vegfabd,c~s) -> vegf(r,r,nrp1bd,c~s) + vegf(r,r,nrp1bd!1,c~s).NRP1(vegfabd!1,c~s) kVEGFNRP1on

vegf(r,r,nrp1bd!1,c~s).NRP1(vegfabd!1,c~s) ->  NRP1(vegfabd,c~s)    kVEGFNRP1off


vegf(r,r,nrp1bd!+,c~s) + vegfr2(l1,dimer,c~s) <-> \

vegf(r!2,r,nrp1bd!+,c~s).vegfr2(l1!2,dimer,c~s) kNRP1VEGFR2on,kNRP1VEGFR2off

vegf(r,r,nrp1bd!+,c~s) + vegfr2(l1,dimer!3,c~s).vegfr2(l1,dimer!3,c~s) <-> \

vegf(r!2,r,nrp1bd!+,c~s).vegfr2(l1!2,dimer!3,c~s).vegfr2(l1,dimer!3,c~s) kNRP1VEGFR2on,kNRP1VEGFR2off

vegf(r,r,nrp1bd!+,c~s) + vegfr2(l1,dimer!3,c~s).vegfr1(l2,dimer!3,c~s) <-> \

vegf(r!2,r,nrp1bd!+,c~s).vegfr2(l1!2,dimer!3,c~s).vegfr1(l2,dimer!3,c~s) kNRP1VEGFR2on,kNRP1VEGFR2off


vegf(r,r,nrp1bd!+,c~s) + vegfr1(l2,dimer,c~s) <-> \

vegf(r!2,r,nrp1bd!+,c~s).vegfr1(l2!2,dimer,c~s) kcVR,kvr1off

vegf(r,r,nrp1bd!+,c~s) + vegfr1(l2,dimer!3,c~s).vegfr1(l2,dimer!3,c~s) <-> \

vegf(r!2,r,nrp1bd!+,c~s).vegfr1(l2!2,dimer!3,c~s).vegfr1(l2,dimer!3,c~s) kcVR,kvr1off

vegf(r,r,nrp1bd!+,c~s) + vegfr1(l2,dimer!3,c~s).vegfr2(l1,dimer!3,c~s) <-> \

vegf(r!2,r,nrp1bd!+,c~s).vegfr1(l2!2,dimer!3,c~s).vegfr2(l1,dimer!3,c~s) kcVR,kvr1off


# Binding of VEGF165a to VEGFR2 receptors


I() -> I() + vegf(r,r,nrp1bd,c~s)     -(kvron*cellarea/fextmolar)*vegffrees*vr2s+(kvroff*cellarea/fextmolar)*vegfr2s

vegf(r,r,nrp1bd,c~s) + vegfr2(l1,c~s) -> \

vegf(r,r,nrp1bd,c~s) + vegf(r!1,r,nrp1bd,c~s).vegfr2(l1!1,c~s) kvron

vegf(r!1,r,nrp1bd,c~s).vegfr2(l1!1,c~s) -> vegfr2(l1,c~s) kvroff


vegf(r!1,r,nrp1bd,c~s).vegfr2(l1!1,dimer,c~s) + vegfr2(l1,dimer,c~s) <-> \
```

```
vegf(r!1,r!2,nrp1bd,c~s).vegfr2(l1!1,dimer,c~s).vegfr2(l1!2,dimer,c~s) kcVR,kvroff

vegf(r!1,r,nrp1bd,c~s).vegfr2(l1!1,dimer,c~s) + vegfr1(l2,dimer,c~s) <-> \

vegf(r!1,r!2,nrp1bd,c~s).vegfr2(l1!1,dimer,c~s).vegfr1(l2!2,dimer,c~s) kcVR,kvr1off


vegf(r!1,r,nrp1bd,c~s).vegfr2(l1!1,dimer!3,c~s).vegfr2(l1,dimer!3,c~s) <-> \

vegf(r!1,r!2,nrp1bd,c~s).vegfr2(l1!1,dimer!3,c~s).vegfr2(l1!2,dimer!3,c~s) kdeltaVR,kvroff

vegf(r!1,r,nrp1bd,c~s).vegfr2(l1!1,dimer!3,c~s).vegfr1(l2,dimer!3,c~s) <-> \

vegf(r!1,r!2,nrp1bd,c~s).vegfr2(l1!1,dimer!3,c~s).vegfr1(l2!2,dimer!3,c~s) kdeltaVR,kvr1off


vegf(r!1,r,nrp1bd,c~s).vegfr1(l2!1,dimer,c~s) + vegfr1(l2,dimer,c~s) <-> \

vegf(r!1,r!2,nrp1bd,c~s).vegfr1(l2!1,dimer,c~s).vegfr1(l2!2,dimer,c~s) kcVR,kvr1off

vegf(r!1,r,nrp1bd,c~s).vegfr1(l2!1,dimer,c~s) + vegfr2(l1,dimer,c~s) <-> \

vegf(r!1,r!2,nrp1bd,c~s).vegfr1(l2!1,dimer,c~s).vegfr2(l1!2,dimer,c~s) kcVR,kvroff

vegf(r!1,r,nrp1bd,c~s).vegfr1(l2!1,dimer!3,c~s).vegfr1(l2,dimer!3,c~s) <-> \

vegf(r!1,r!2,nrp1bd,c~s).vegfr1(l2!1,dimer!3,c~s).vegfr1(l2!2,dimer!3,c~s) kdeltaVR,kvr1off

vegf(r!1,r,nrp1bd,c~s).vegfr1(l2!1,dimer!3,c~s).vegfr2(l1,dimer!3,c~s) <-> \

vegf(r!1,r!2,nrp1bd,c~s).vegfr1(l2!1,dimer!3,c~s).vegfr2(l1!2,dimer!3,c~s) kdeltaVR,kvroff


vegf(r!1,r,nrp1bd!+,c~s).vegfr2(l1!1,dimer,c~s) + vegfr2(l1,dimer,c~s) <-> \

vegf(r!1,r!2,nrp1bd!+,c~s).vegfr2(l1!1,dimer,c~s).vegfr2(l1!2,dimer,c~s) kNRP1VEGFR2on,kNRP1VEGFR2off

vegf(r!1,r,nrp1bd!+,c~s).vegfr2(l1!1,dimer,c~s) + vegfr1(l2,dimer,c~s) <-> \

vegf(r!1,r!2,nrp1bd!+,c~s).vegfr2(l1!1,dimer,c~s).vegfr1(l2!2,dimer,c~s) kcVR,kvr1off


vegf(r!1,r,nrp1bd!+,c~s).vegfr2(l1!1,dimer!3,c~s).vegfr2(l1,dimer!3,c~s) <-> \

vegf(r!1,r!2,nrp1bd!+,c~s).vegfr2(l1!1,dimer!3,c~s).vegfr2(l1!2,dimer!3,c~s) kdeltaVR,kvroff

vegf(r!1,r,nrp1bd!+,c~s).vegfr2(l1!1,dimer!3,c~s).vegfr1(l2,dimer!3,c~s) <-> \

vegf(r!1,r!2,nrp1bd!+,c~s).vegfr2(l1!1,dimer!3,c~s).vegfr1(l2!2,dimer!3,c~s) kdeltaVR,kvr1off

vegf(r!1,r,nrp1bd!+,c~s).vegfr1(l2!1,dimer,c~s) + vegfr1(l2,dimer,c~s) <-> \

vegf(r!1,r!2,nrp1bd!+,c~s).vegfr1(l2!1,dimer,c~s).vegfr1(l2!2,dimer,c~s) kcVR,kvr1off


vegf(r!1,r,nrp1bd!+,c~s).vegfr1(l2!1,dimer,c~s) + vegfr2(l1,dimer,c~s) <-> \
```

```
vegf(r!1,r!2,nrp1bd!+,c~s).vegfr1(l2!1,dimer,c~s).vegfr2(l1!2,dimer,c~s) kNRP1VEGFR2on,kNRP1VEGFR2off

vegf(r!1,r,nrp1bd!+,c~s).vegfr1(l2!1,dimer!3,c~s).vegfr1(l2,dimer!3,c~s) <-> \

vegf(r!1,r!2,nrp1bd!+,c~s).vegfr1(l2!1,dimer!3,c~s).vegfr1(l2!2,dimer!3,c~s) kdeltaVR,kvr1off

vegf(r!1,r,nrp1bd!+,c~s).vegfr1(l2!1,dimer!3,c~s).vegfr2(l1,dimer!3,c~s) <-> \

vegf(r!1,r!2,nrp1bd!+,c~s).vegfr1(l2!1,dimer!3,c~s).vegfr2(l1!2,dimer!3,c~s) kdeltaVR,kvroff


vegf(r!1,r!2,nrp1bd,c~s).vegfr2(l1!1,dimer!3,c~s).vegfr2(l1!2,dimer!3,c~s) + NRP1(vegfabd,c~s)  <-> \

vegf(r!1,r!2,nrp1bd!4,c~s).vegfr2(l1!1,dimer!3,c~s).vegfr2(l1!2,dimer!3,c~s).NRP1(vegfabd!4,c~s)
kVEGFR2NRP1on,kVEGFR2NRP1off


vegf(r!1,r!2,nrp1bd,c~s).vegfr1(l2!1,dimer!3,c~s).vegfr1(l2!2,dimer!3,c~s) + NRP1(vegfabd,c~s)  <-> \

vegf(r!1,r!2,nrp1bd!4,c~s).vegfr1(l2!1,dimer!3,c~s).vegfr1(l2!2,dimer!3,c~s).NRP1(vegfabd!4,c~s)
kVEGFR2NRP1on,kVEGFR2NRP1off


vegf(r!1,r!2,nrp1bd,c~s).vegfr2(l1!1,dimer!3,c~s).vegfr1(l2!2,dimer!3,c~s) + NRP1(vegfabd,c~s)  <-> \

vegf(r!1,r!2,nrp1bd!4,c~s).vegfr2(l1!1,dimer!3,c~s).vegfr1(l2!2,dimer!3,c~s).NRP1(vegfabd!4,c~s)
kVEGFR2NRP1on,kVEGFR2NRP1off


vegf(r!1,r!2,nrp1bd,c~s).vegfr2(l1!1,dimer,c~s).vegfr2(l1!2,dimer,c~s) + NRP1(vegfabd,c~s)  <-> \

vegf(r!1,r!2,nrp1bd!4,c~s).vegfr2(l1!1,dimer,c~s).vegfr2(l1!2,dimer,c~s).NRP1(vegfabd!4,c~s)
kVEGFR2NRP1on,kVEGFR2NRP1off

vegf(r!1,r,nrp1bd,c~s).vegfr2(l1!1,c~s) + NRP1(vegfabd,c~s) <-> vegf(r!1,r,nrp1bd!2,c~s).vegfr2(l1!1,c~s).NRP1(vegfabd!2,c~s)
kVEGFR2NRP1on,kVEGFR2NRP1off

vegf(r!1,r,nrp1bd,c~s).vegfr1(l2!1,c~s) + NRP1(vegfabd,c~s) <-> vegf(r!1,r,nrp1bd!2,c~s).vegfr1(l2!1,c~s).NRP1(vegfabd!2,c~s)
kVEGFR1NRP1on,kVEGFR1NRP1off


#######################


# vegf165a binding to vegfr1


I() -> I() + vegf(r,r,nrp1bd,c~s)   -kvr1on*cellarea/fextmolar*vegffrees*vr1s+kvr1off*vegfr1s*(cellarea/fextmolar)

vegf(r,r,nrp1bd,c~s) + vegfr1(l2,c~s) -> \

vegf(r,r,nrp1bd,c~s) + vegf(r!1,r,nrp1bd,c~s).vegfr1(l2!1,c~s) kvron

vegf(r!1,r,nrp1bd,c~s).vegfr1(l2!1,c~s) -> vegfr1(l2,c~s) kvr1off
```

```
vegf(r!1,r,c~s).vegfr1(l2!1,dimer,c~s) + vegfr1(l2,dimer,c~s) <-> \

vegf(r!1,r!2,c~s).vegfr1(l2!1,dimer,c~s).vegfr1(l2!2,dimer,c~s) kcVR,kvr1off

vegf(r!1,r,c~s).vegfr1(l2!1,dimer,c~s) + vegfr2(l1,dimer,c~s) <-> \

vegf(r!1,r!2,c~s).vegfr1(l2!1,dimer,c~s).vegfr2(l1!2,dimer,c~s) kcVR,kvroff

vegf(r!1,r,c~s).vegfr1(l2!1,dimer!3,c~s).vegfr1(l2,dimer!3,c~s) <-> \

vegf(r!1,r!2,c~s).vegfr1(l2!1,dimer!3,c~s).vegfr1(l2!2,dimer!3,c~s) kdeltaVR,kvr1off

vegf(r!1,r,c~s).vegfr1(l2!1,dimer!3,c~s).vegfr2(l1,dimer!3,c~s) <-> \

vegf(r!1,r!2,c~s).vegfr1(l2!1,dimer!3,c~s).vegfr2(l1!2,dimer!3,c~s) kdeltaVR,kvroff


################################


# Receptor phosphorylation


vegf(r!1,r!2,c~s).vegfr2(l1!1,c~s).vegfr2(l1!2,Y1175~Y,c~s) -> \

vegf(r!1,r!2,c~s).vegfr2(l1!1,c~s).vegfr2(l1!2,Y1175~pY,c~s) kpY1175


vegf(r!1,r!2,c~i).vegfr2(l1!1,c~i).vegfr2(l1!2,Y1175~Y,c~i) -> \

vegf(r!1,r!2,c~i).vegfr2(l1!1,c~i).vegfr2(l1!2,Y1175~pY,c~i)  kpY1175


# Dephosphorylation of VEGFR2 species


vegfr2(Y1175~pY,CD47bd!1,c~s).CD47SIRPa(TSP1bd!+,VEGFR2bd!1,c~s) ->
vegfr2(Y1175~Y,CD47bd!1,c~s).CD47SIRPa(TSP1bd!+,VEGFR2bd!1,c~s)  kdpTSP1s

vegfr2(Y1175~pY,CD47bd!1,c~i).CD47SIRPa(TSP1bd!+,VEGFR2bd!1,c~i) ->
vegfr2(Y1175~Y,CD47bd!1,c~i).CD47SIRPa(TSP1bd!+,VEGFR2bd!1,c~i)  kdpTSP1i


vegfr2(Y1175~pY,CD47bd!1,c~s).CD47SIRPa(TSP1bd,VEGFR2bd!1,c~s) ->
vegfr2(Y1175~Y,CD47bd!1,c~s).CD47SIRPa(TSP1bd,VEGFR2bd!1,c~s)  kdps

vegfr2(Y1175~pY,CD47bd!1,c~i).CD47SIRPa(TSP1bd,VEGFR2bd!1,c~i) ->
vegfr2(Y1175~Y,CD47bd!1,c~i).CD47SIRPa(TSP1bd,VEGFR2bd!1,c~i)  kdpi


vegfr2(Y1175~pY,CD47bd,c~s) -> vegfr2(Y1175~Y,CD47bd,c~s)  kdps

vegfr2(Y1175~pY,CD47bd,c~i) -> vegfr2(Y1175~Y,CD47bd,c~i)  kdpi
```

# Internalization s to i

**vegf(r!1,r!2,nrp1bd,c~s).vegfr2(l1!1,dimer,CD47bd,c~s).vegfr2(l1!2,CD47bd,dimer,c~s) -> **

vegf(r!1,r!2,nrp1bd,c~i).vegfr2(l1!1,dimer,CD47bd,c~i).vegfr2(l1!2,dimer,CD47bd,c~i) kr2si

**vegf(r!1,r!2,nrp1bd,c~s).vegfr2(l1!1,dimer!6,CD47bd,c~s).vegfr2(l1!2,dimer!6,CD47bd,c~s) -> **

vegf(r!1,r!2,nrp1bd,c~i).vegfr2(l1!1,dimer!6,CD47bd,c~i).vegfr2(l1!2,dimer!6,CD47bd,c~i) kr2si

**vegf(r!1,r!2,nrp1bd,c~s).vegfr2(l1!1,dimer,CD47bd!3,c~s).CD47SIRPa(TSP1bd,VEGFR2bd!3,c~s).vegfr2(l1!2,CD47bd,dimer,c~s) -> **

vegf(r!1,r!2,nrp1bd,c~i).vegfr2(l1!1,dimer,CD47bd!3,c~i).CD47SIRPa(TSP1bd,VEGFR2bd!3,c~i).vegfr2(l1!2,dimer,CD47bd,c~i) kr2si

**vegf(r!1,r!2,nrp1bd,c~s).vegfr2(l1!1,dimer!6,CD47bd!3,c~s).CD47SIRPa(TSP1bd,VEGFR2bd!3,c~s).vegfr2(l1!2,dimer!6,CD47bd,c~s) -> **

vegf(r!1,r!2,nrp1bd,c~i).vegfr2(l1!1,dimer!6,CD47bd!3,c~i).CD47SIRPa(TSP1bd,VEGFR2bd!3,c~i).vegfr2(l1!2,dimer!6,CD47bd,c~i) kr2si

**vegf(r!1,r!2,nrp1bd,c~s).vegfr2(l1!1,dimer,CD47bd!3,c~s).CD47SIRPa(TSP1bd!+,VEGFR2bd!3,c~s).vegfr2(l1!2,CD47bd,dimer,c~s) -> **

vegf(r!1,r!2,nrp1bd,c~i).vegfr2(l1!1,dimer,CD47bd!3,c~i).CD47SIRPa(TSP1bd!+,VEGFR2bd!3,c~i).vegfr2(l1!2,dimer,CD47bd,c~i) kr2TSP1si

**vegf(r!1,r!2,nrp1bd,c~s).vegfr2(l1!1,dimer!6,CD47bd!3,c~s).CD47SIRPa(TSP1bd!+,VEGFR2bd!3,c~s).vegfr2(l1!2,dimer!6,CD47bd,c~s) -> **

vegf(r!1,r!2,nrp1bd,c~i).vegfr2(l1!1,dimer!6,CD47bd!3,c~i).CD47SIRPa(TSP1bd!+,VEGFR2bd!3,c~i).vegfr2(l1!2,dimer!6,CD47bd,c~i) kr2TSP1si

**vegf(r!1,r!2,nrp1bd,c~s).vegfr2(l1!1,dimer,CD47bd!4,c~s).CD47SIRPa(TSP1bd,VEGFR2bd!4,c~s).vegfr2(l1!2,dimer,CD47bd!3,c~s).CD47SIRPa(TSP1bd,VEGFR2bd!3,c~s) -> **

vegf(r!1,r!2,nrp1bd,c~i).vegfr2(l1!1,dimer,CD47bd!4,c~i).CD47SIRPa(TSP1bd,VEGFR2bd!4,c~i).vegfr2(l1!2,dimer,CD47bd!3,c~i).CD47SIRPa(TSP1bd,VEGFR2bd!3,c~i) kr2si

**vegf(r!1,r!2,nrp1bd,c~s).vegfr2(l1!1,dimer!6,CD47bd!4,c~s).CD47SIRPa(TSP1bd,VEGFR2bd!4,c~s).vegfr2(l1!2,dimer!6,CD47bd!3,c~s).CD47SIRPa(TSP1bd,VEGFR2bd!3,c~s) -> **

vegf(r!1,r!2,nrp1bd,c~i).vegfr2(l1!1,dimer!6,CD47bd!4,c~i).CD47SIRPa(TSP1bd,VEGFR2bd!4,c~i).vegfr2(l1!2,dimer!6,CD47bd!3,c~i).CD47SIRPa(TSP1bd,VEGFR2bd!3,c~i) kr2si

**vegf(r!1,r!2,nrp1bd,c~s).vegfr2(l1!1,dimer,CD47bd!4,c~s).CD47SIRPa(TSP1bd!+,VEGFR2bd!4,c~s).vegfr2(l1!2,dimer,CD47bd!3,c~s).CD47SIRPa(TSP1bd!+,VEGFR2bd!3,c~s) -> **

vegf(r!1,r!2,nrp1bd,c~i).vegfr2(l1!1,dimer,CD47bd!4,c~i).CD47SIRPa(TSP1bd!+,VEGFR2bd!4,c~i).vegfr2(l1!2,dimer,CD47bd!3,c~i).CD47SIRPa(TSP1bd!+,VEGFR2bd!3,c~i) kr2TSP1si

vegf(r!1,r!2,nrp1bd,c~s).vegfr2(l1!1,dimer!6,CD47bd!4,c~s).CD47SIRPa(TSP1bd!+,VEGFR2bd!4,c~s).vegfr2(l1!2,dimer!6,CD47bd!3,c~s).CD47SIRPa(TSP1bd!+,VEGFR2bd!3,c~s) -> \

vegf(r!1,r!2,nrp1bd,c~i).vegfr2(l1!1,dimer!6,CD47bd!4,c~i).CD47SIRPa(TSP1bd!+,VEGFR2bd!4,c~i).vegfr2(l1!2,dimer!6,CD47bd!3,c~i).CD47SIRPa(TSP1bd!+,VEGFR2bd!3,c~i) kr2TSP1si

vegf(r!1,r!2,nrp1bd!9,c~s).NRP1(vegfabd!9,c~s).vegfr2(l1!1,dimer,CD47bd,c~s).vegfr2(l1!2,dimer,CD47bd,c~s) -> \

vegf(r!1,r!2,nrp1bd!9,c~i).NRP1(vegfabd!9,c~i).vegfr2(l1!1,dimer,CD47bd,c~i).vegfr2(l1!2,dimer,CD47bd,c~i) kr2NRP1si

vegf(r!1,r!2,nrp1bd!9,c~s).NRP1(vegfabd!9,c~s).vegfr2(l1!1,dimer!6,CD47bd,c~s).vegfr2(l1!2,dimer!6,CD47bd,c~s) -> \

vegf(r!1,r!2,nrp1bd!9,c~i).NRP1(vegfabd!9,c~i).vegfr2(l1!1,dimer!6,CD47bd,c~i).vegfr2(l1!2,dimer!6,CD47bd,c~i) kr2NRP1si

vegf(r!1,r!2,nrp1bd!9,c~s).NRP1(vegfabd!9,c~s).vegfr2(l1!1,dimer,CD47bd!3,c~s).CD47SIRPa(TSP1bd,VEGFR2bd!3,c~s).vegfr2(l1!2,CD47bd,dimer,c~s) -> \

vegf(r!1,r!2,nrp1bd!9,c~i).NRP1(vegfabd!9,c~i).vegfr2(l1!1,dimer,CD47bd!3,c~i).CD47SIRPa(TSP1bd,VEGFR2bd!3,c~i).vegfr2(l1!2,dimer,CD47bd,c~i) kr2NRP1si

vegf(r!1,r!2,nrp1bd!9,c~s).NRP1(vegfabd!9,c~s).vegfr2(l1!1,dimer!6,CD47bd!3,c~s).CD47SIRPa(TSP1bd,VEGFR2bd!3,c~s).vegfr2(l1!2,dimer!6,CD47bd,c~s) -> \

vegf(r!1,r!2,nrp1bd!9,c~i).NRP1(vegfabd!9,c~i).vegfr2(l1!1,dimer!6,CD47bd!3,c~i).CD47SIRPa(TSP1bd,VEGFR2bd!3,c~i).vegfr2(l1!2,dimer!6,CD47bd,c~i) kr2NRP1si

vegf(r!1,r!2,nrp1bd!9,c~s).NRP1(vegfabd!9,c~s).vegfr2(l1!1,dimer,CD47bd!3,c~s).CD47SIRPa(TSP1bd!+,VEGFR2bd!3,c~s).vegfr2(l1!2,CD47bd,dimer,c~s) -> \

vegf(r!1,r!2,nrp1bd!9,c~i).NRP1(vegfabd!9,c~i).vegfr2(l1!1,dimer,CD47bd!3,c~i).CD47SIRPa(TSP1bd!+,VEGFR2bd!3,c~i).vegfr2(l1!2,dimer,CD47bd,c~i) kr2NRP1TSP1si

vegf(r!1,r!2,nrp1bd!9,c~s).NRP1(vegfabd!9,c~s).vegfr2(l1!1,dimer!6,CD47bd!3,c~s).CD47SIRPa(TSP1bd!+,VEGFR2bd!3,c~s).vegfr2(l1!2,dimer!6,CD47bd,c~s) -> \

vegf(r!1,r!2,nrp1bd!9,c~i).NRP1(vegfabd!9,c~i).vegfr2(l1!1,dimer!6,CD47bd!3,c~i).CD47SIRPa(TSP1bd!+,VEGFR2bd!3,c~i).vegfr2(l1!2,dimer!6,CD47bd,c~i) kr2NRP1TSP1si

vegf(r!1,r!2,nrp1bd!9,c~s).NRP1(vegfabd!9,c~s).vegfr2(l1!1,dimer,CD47bd!4,c~s).CD47SIRPa(TSP1bd,VEGFR2bd!4,c~s).vegfr2(l1!2,dimer,CD47bd!3,c~s).CD47SIRPa(TSP1bd,VEGFR2bd!3,c~s) -> \

vegf(r!1,r!2,nrp1bd!9,c~i).NRP1(vegfabd!9,c~i).vegfr2(l1!1,dimer,CD47bd!4,c~i).CD47SIRPa(TSP1bd,VEGFR2bd!4,c~i).vegfr2(l1!2,dimer,CD47bd!3,c~i).CD47SIRPa(TSP1bd,VEGFR2bd!3,c~i) kr2NRP1si

vegf(r!1,r!2,nrp1bd!9,c~s).NRP1(vegfabd!9,c~s).vegfr2(l1!1,dimer!6,CD47bd!4,c~s).CD47SIRPa(TSP1bd,VEGFR2bd!4,c~s).vegfr2(l1!2,dimer!6,CD47bd!3,c~s).CD47SIRPa(TSP1bd,VEGFR2bd!3,c~s) -> \

vegf(r!1,r!2,nrp1bd!9,c~i).NRP1(vegfabd!9,c~i).vegfr2(l1!1,dimer!6,CD47bd!4,c~i).CD47SIRPa(TSP1bd,VEGFR2bd!4,c~i).vegfr2(l1!2,dimer!6,CD47bd!3,c~i).CD47SIRPa(TSP1bd,VEGFR2bd!3,c~i) kr2NRP1si

vegf(r!1,r!2,nrp1bd!9,c~s).NRP1(vegfabd!9,c~s).vegfr2(l1!1,dimer,CD47bd!4,c~s).CD47SIRPa(TSP1bd!+,VEGFR2bd!4,c~s).vegfr2(l1!2,dimer,CD47bd!3,c~s).CD47SIRPa(TSP1bd!+,VEGFR2bd!3,c~s) -> \

vegf(r!1,r!2,nrp1bd!9,c~i).NRP1(vegfabd!9,c~i).vegfr2(l1!1,dimer,CD47bd!4,c~i).CD47SIRPa(TSP1bd!+,VEGFR2bd!4,c~i).vegfr2(l1!2,dimer,CD47bd!3,c~i).CD47SIRPa(TSP1bd!+,VEGFR2bd!3,c~i) kr2NRP1TSP1si

vegf(r!1,r!2,nrp1bd!9,c~s).NRP1(vegfabd!9,c~s).vegfr2(l1!1,dimer!6,CD47bd!4,c~s).CD47SIRPa(TSP1bd!+,VEGFR2bd!4,c~s).vegfr2(l1!2,dimer!6,CD47bd!3,c~s).CD47SIRPa(TSP1bd!+,VEGFR2bd!3,c~s) -> \

vegf(r!1,r!2,nrp1bd!9,c~i).NRP1(vegfabd!9,c~i).vegfr2(l1!1,dimer!6,CD47bd!4,c~i).CD47SIRPa(TSP1bd!+,VEGFR2bd!4,c~i).vegfr2(l1!2,dimer!6,CD47bd!3,c~i).CD47SIRPa(TSP1bd!+,VEGFR2bd!3,c~i) kr2NRP1TSP1si

Recycling i to s

vegf(r!1,r!2,nrp1bd,c~i).vegfr2(l1!1,dimer,CD47bd,c~i).vegfr2(l1!2,dimer,CD47bd,c~i) -> \

vegf(r!1,r!2,nrp1bd,c~s).vegfr2(l1!1,dimer,CD47bd,c~s).vegfr2(l1!2,dimer,CD47bd,c~s) kr2is

vegf(r!1,r!2,nrp1bd,c~i).vegfr2(l1!1,dimer!6,CD47bd,c~i).vegfr2(l1!2,dimer!6,CD47bd,c~i) -> \

vegf(r!1,r!2,nrp1bd,c~s).vegfr2(l1!1,dimer!6,CD47bd,c~s).vegfr2(l1!2,dimer!6,CD47bd,c~s) kr2is

vegf(r!1,r!2,nrp1bd,c~i).vegfr2(l1!1,dimer,CD47bd!3,c~i).CD47SIRPa(VEGFR2bd!3,c~i).vegfr2(l1!2,dimer,CD47bd,c~i) -> \

vegf(r!1,r!2,nrp1bd,c~s).vegfr2(l1!1,dimer,CD47bd!3,c~s).CD47SIRPa(VEGFR2bd!3,c~s).vegfr2(l1!2,dimer,CD47bd,c~s) kr2is

vegf(r!1,r!2,nrp1bd,c~i).vegfr2(l1!1,dimer!6,CD47bd!3,c~i).CD47SIRPa(VEGFR2bd!4,c~i).vegfr2(l1!2,dimer!6,CD47bd,c~i) -> \

vegf(r!1,r!2,nrp1bd,c~s).vegfr2(l1!1,dimer!6,CD47bd!3,c~s).CD47SIRPa(VEGFR2bd!4,c~s).vegfr2(l1!2,dimer!6,CD47bd,c~s) kr2is

vegf(r!1,r!2,nrp1bd,c~i).vegfr2(l1!1,dimer,CD47bd!4,c~i).CD47SIRPa(VEGFR2bd!4,c~i).vegfr2(l1!2,dimer,CD47bd!3,c~i).CD47SIRPa(VEGFR2bd!3,c~i) -> \

vegf(r!1,r!2,nrp1bd,c~s).vegfr2(l1!1,dimer,CD47bd!4,c~s).CD47SIRPa(VEGFR2bd!4,c~s).vegfr2(l1!2,dimer,CD47bd!3,c~s).CD47SIRPa(VEGFR2bd!3,c~s) kr2is

vegf(r!1,r!2,nrp1bd,c~i).vegfr2(l1!1,dimer!6,CD47bd!4,c~i).CD47SIRPa(VEGFR2bd!4,c~i).vegfr2(l1!2,dimer!6,CD47bd!3,c~i).CD47SIRPa(VEGFR2bd!3,c~i) -> \

vegf(r!1,r!2,nrp1bd,c~s).vegfr2(l1!1,dimer!6,CD47bd!4,c~s).CD47SIRPa(VEGFR2bd!4,c~s).vegfr2(l1!2,dimer!6,CD47bd!3,c~s).CD47SIRPa(VEGFR2bd!3,c~s) kr2is

vegf(r!1,r!2,nrp1bd!9,c~i).NRP1(vegfabd!9,c~i).vegfr2(l1!1,dimer,CD47bd,c~i).vegfr2(l1!2,dimer,CD47bd,c~i) -> \

vegf(r!1,r!2,nrp1bd!9,c~s).NRP1(vegfabd!9,c~s).vegfr2(l1!1,dimer,CD47bd,c~s).vegfr2(l1!2,dimer,CD47bd,c~s) kr2NRP1is

vegf(r!1,r!2,nrp1bd!9,c~i).NRP1(vegfabd!9,c~i).vegfr2(l1!1,dimer!6,CD47bd,c~i).vegfr2(l1!2,dimer!6,CD47bd,c~i) -> \

vegf(r!1,r!2,nrp1bd!9,c~s).NRP1(vegfabd!9,c~s).vegfr2(l1!1,dimer!6,CD47bd,c~s).vegfr2(l1!2,dimer!6,CD47bd,c~s) kr2NRP1is

vegf(r!1,r!2,nrp1bd!9,c~i).NRP1(vegfabd!9,c~i).vegfr2(l1!1,dimer,CD47bd!3,c~i).CD47SIRPa(VEGFR2bd!3,c~i).vegfr2(l1!2,dimer,CD47bd,c~i) -> \

vegf(r!1,r!2,nrp1bd!9,c~s).NRP1(vegfabd!9,c~s).vegfr2(l1!1,dimer,CD47bd!3,c~s).CD47SIRPa(VEGFR2bd!3,c~s).vegfr2(l1!2,dimer,CD47bd,c~s) kr2NRP1is

vegf(r!1,r!2,nrp1bd!9,c~i).NRP1(vegfabd!9,c~i).vegfr2(l1!1,dimer!6,CD47bd!3,c~i).CD47SIRPa(VEGFR2bd!4,c~i).vegfr2(l1!2,dimer!6,CD47bd,c~i) -> \

vegf(r!1,r!2,nrp1bd!9,c~s).NRP1(vegfabd!9,c~s).vegfr2(l1!1,dimer!6,CD47bd!3,c~s).CD47SIRPa(VEGFR2bd!4,c~s).vegfr2(l1!2,dimer!6,CD47bd,c~s) kr2NRP1is

vegf(r!1,r!2,nrp1bd!9,c~i).NRP1(vegfabd!9,c~i).vegfr2(l1!1,dimer,CD47bd!4,c~i).CD47SIRPa(VEGFR2bd!4,c~i).vegfr2(l1!2,dimer,CD47bd!3,c~i).CD47SIRPa(VEGFR2bd!3,c~i) -> \

vegf(r!1,r!2,nrp1bd!9,c~s).NRP1(vegfabd!9,c~s).vegfr2(l1!1,dimer,CD47bd!4,c~s).CD47SIRPa(VEGFR2bd!4,c~s).vegfr2(l1!2,dimer,CD47bd!3,c~s).CD47SIRPa(VEGFR2bd!3,c~s) kr2NRP1is

vegf(r!1,r!2,nrp1bd!9,c~i).NRP1(vegfabd!9,c~i).vegfr2(l1!1,dimer!6,CD47bd!4,c~i).CD47SIRPa(VEGFR2bd!4,c~i).vegfr2(l1!2,dimer!6,CD47bd!3,c~i).CD47SIRPa(VEGFR2bd!3,c~i) -> \

vegf(r!1,r!2,nrp1bd!9,c~s).NRP1(vegfabd!9,c~s).vegfr2(l1!1,dimer!6,CD47bd!4,c~s).CD47SIRPa(VEGFR2bd!4,c~s).vegfr2(l1!2,dimer!6,CD47bd!3,c~s).CD47SIRPa(VEGFR2bd!3,c~s) kr2NRP1is

##

vegfr2(l1,dimer,CD47bd!1,c~i).CD47SIRPa(VEGFR2bd!1,c~i) -> vegfr2(l1,dimer,CD47bd!1,c~s).CD47SIRPa(VEGFR2bd!1,c~s) kr2isnoUB

vegfr2(l1,Y1175~Y,CD47bd!1,dimer!3,c~i).CD47SIRPa(VEGFR2bd!1,c~i).vegfr2(l1,Y1175~Y,CD47bd!2,dimer!3,c~i).CD47SIRPa(VEGFR2bd!2,c~i) \

->
vegfr2(l1,Y1175~Y,CD47bd!1,dimer!3,c~s).CD47SIRPa(VEGFR2bd!1,c~s).vegfr2(l1,Y1175~Y,CD47bd!2,dimer!3,c~s).CD47SIRPa(VEGFR2bd!2,c~s) kr2isnoUB

vegfr2(l1,dimer,CD47bd,c~i) -> vegfr2(l1,dimer,CD47bd,c~s)   kr2isnoUB

vegfr2(l1,Y1175~Y,CD47bd,dimer!3,c~i).vegfr2(l1,Y1175~Y,CD47bd,dimer!3,c~i) \

-> vegfr2(l1,Y1175~Y,CD47bd,dimer!3,c~s).vegfr2(l1,Y1175~Y,CD47bd,dimer!3,c~s) kr2isnoUB

##

Receptor degradation

# Free VEGFR2

vegf(r!1,r!2,nrp1bd,c~i).vegfr2(l1!1,CD47bd,Y1175~pY,c~i).vegfr2(l1!2,CD47bd,c~i) -> Trash() kdegi0

vegf(r!1,r!2,nrp1bd,c~i).vegfr2(l1!1,CD47bd,Y1175~Y,c~i).vegfr2(l1!2,CD47bd,,Y1175~Y,c~i) -> Trash() kdegi0noUB

vegfr2(l1,CD47bd,dimer,c~i) -> Trash() kdegi0noUB

vegfr2(l1,CD47bd,dimer!1,c~i).vegfr2(l1,CD47bd,dimer!1,c~i) -> Trash() kdegi0noUB

vegf(r!1,r!2,nrp1bd!+,c~i).vegfr2(l1!1,CD47bd,Y1175~pY,c~i).vegfr2(l1!2,CD47bd,c~i) -> Trash() kdegr2NRP1i0

vegf(r!1,r!2,nrp1bd!+,c~i).vegfr2(l1!1,CD47bd,Y1175~Y,c~i).vegfr2(l1!2,CD47bd,Y1175~Y,c~i) -> Trash() kdegr2NRP1i0noUB

# VEGFR2/CD47 no TSP1

vegf(r!1,r!2,nrp1bd,c~i).vegfr2(l1!1,CD47bd!3,Y1175~pY,c~i).vegfr2(l1!2,CD47bd,c~i).CD47SIRPa(TSP1bd,VEGFR2bd!3,c~i) -> Trash() kdegi0

vegf(r!1,r!2,nrp1bd,c~i).vegfr2(l1!1,CD47bd,Y1175~pY,c~i).vegfr2(l1!2,CD47bd!3,c~i).CD47SIRPa(TSP1bd,VEGFR2bd!3,c~i) -> Trash() kdegi0

**vegf(r!1,r!2,nrp1bd,c~i).vegfr2(l1!1,CD47bd!3,Y1175~pY,c~i).vegfr2(l1!2,CD47bd!4,c~i).CD47SIRPa(TSP1bd,VEGFR2bd!4,c~i).CD47SIRPa(TSP1bd,VEGFR2bd!3,c~i) -> **

Trash() kdegi0

vegf(r!1,r!2,nrp1bd,c~i).vegfr2(l1!1,CD47bd!3,Y1175~Y,c~i).vegfr2(l1!2,CD47bd,Y1175~Y,c~i).CD47SIRPa(TSP1bd,VEGFR2bd!3,c~i) -> Trash() kdegi0noUB

**vegf(r!1,r!2,nrp1bd,c~i).vegfr2(l1!1,CD47bd!3,Y1175~Y,c~i).vegfr2(l1!2,CD47bd!4,Y1175~Y,c~i).CD47SIRPa(TSP1bd,VEGFR2bd!3,c~i).CD47SIRPa(TSP1bd,VEGFR2bd!4,c~i) -> **

Trash() kdegi0noUB

vegfr2(l1,CD47bd!1,dimer,c~i).CD47SIRPa(TSP1bd,VEGFR2bd!1,c~i) -> Trash() kdegi0noUB

vegfr2(l1,CD47bd!1,dimer!2,c~i).vegfr2(l1,CD47bd,dimer!2,c~i).CD47SIRPa(TSP1bd,VEGFR2bd!1,c~i) -> Trash() kdegi0noUB

vegfr2(l1,CD47bd!1,dimer!3,c~i).vegfr2(l1,CD47bd!2,dimer!3,c~i).CD47SIRPa(TSP1bd,VEGFR2bd!1,c~i).CD47SIRPa(TSP1bd,VEGFR2bd!2,c~i) -> Trash() kdegi0noUB

vegf(r!1,r!2,nrp1bd!+,c~i).vegfr2(l1!1,CD47bd!3,Y1175~pY,c~i).vegfr2(l1!2,CD47bd,c~i).CD47SIRPa(TSP1bd,VEGFR2bd!3,c~i) -> Trash() kdegr2NRP1i0

vegf(r!1,r!2,nrp1bd!+,c~i).vegfr2(l1!1,CD47bd,Y1175~pY,c~i).vegfr2(l1!2,CD47bd!3,c~i).CD47SIRPa(TSP1bd,VEGFR2bd!3,c~i) -> Trash() kdegr2NRP1i0

vegf(r!1,r!2,nrp1bd!+,c~i).vegfr2(l1!1,CD47bd!3,Y1175~pY,c~i).vegfr2(l1!2,CD47bd!4,c~i).CD47SIRPa(TSP1bd,VEGFR2bd!3,c~i).CD47SIRPa(TSP1bd,VEGFR2bd!4,c~i) -> Trash() kdegr2NRP1i0

vegf(r!1,r!2,nrp1bd!+,c~i).vegfr2(l1!1,CD47bd!3,Y1175~Y,c~i).vegfr2(l1!2,CD47bd,Y1175~Y,c~i).CD47SIRPa(TSP1bd,VEGFR2bd!3,c~i) -> Trash() kdegr2NRP1i0noUB

vegf(r!1,r!2,nrp1bd!+,c~i).vegfr2(l1!1,CD47bd!3,Y1175~Y,c~i).vegfr2(l1!2,CD47bd!4,Y1175~Y,c~i).CD47SIRPa(TSP1bd,VEGFR2bd!3,c~i).CD47SIRPa(TSP1bd,VEGFR2bd!4,c~i) ->

Trash() kdegr2NRP1i0noUB

# TSP1 bound

vegf(r!1,r!2,nrp1bd,c~i).vegfr2(l1!1,CD47bd!3,Y1175~pY,c~i).vegfr2(l1!2,CD47bd,c~i).CD47SIRPa(TSP1bd!+,VEGFR2bd!3,c~i) -> Trash() kdegTSP1i0

vegf(r!1,r!2,nrp1bd,c~i).vegfr2(l1!1,CD47bd,Y1175~pY,c~i).vegfr2(l1!2,CD47bd!3,c~i).CD47SIRPa(TSP1bd!+,VEGFR2bd!3,c~i) -> Trash() kdegTSP1i0

vegf(r!1,r!2,nrp1bd,c~i).vegfr2(l1!1,CD47bd!3,Y1175~pY,c~i).vegfr2(l1!2,CD47bd!4,c~i).CD47SIRPa(TSP1bd!+,VEGFR2bd!4,c~i).CD47SIRPa(VEGFR2bd!3,c~i) -> Trash() kdegTSP1i0

vegf(r!1,r!2,nrp1bd,c~i).vegfr2(l1!1,CD47bd!3,Y1175~Y,c~i).vegfr2(l1!2,CD47bd,Y1175~Y,c~i).CD47SIRPa(TSP1bd!+,VEGFR2bd!3,c~i) -> Trash() kdegTSP1i0noUB

vegf(r!1,r!2,nrp1bd,c~i).vegfr2(l1!1,CD47bd!3,Y1175~Y,c~i).vegfr2(l1!2,CD47bd!4,Y1175~Y,c~i).CD47SIRPa(TSP1bd!+,VEGFR2bd!3,c~i).CD47SIRPa(VEGFR2bd!4,c~i) ->

Trash() kdegTSP1i0noUB

vegfr2(l1,CD47bd!1,dimer,c~i).CD47SIRPa(TSP1bd!+,VEGFR2bd!1,c~i) -> Trash() kdegTSP1i0noUB

vegfr2(l1,CD47bd!1,dimer!2,c~i).vegfr2(l1,CD47bd,dimer!2,c~i).CD47SIRPa(TSP1bd!+,VEGFR2bd!1,c~i) -> Trash() kdegTSP1i0noUB

vegfr2(l1,CD47bd!1,dimer!3,c~i).vegfr2(l1,CD47bd!2,dimer!3,c~i).CD47SIRPa(TSP1bd!+,VEGFR2bd!1,c~i).CD47SIRPa(VEGFR2bd!2,c~i) -> Trash() kdegTSP1i0noUB

vegfr2(l1,CD47bd!1,dimer,c~i).CD47SIRPa(TSP1bd!+,VEGFR2bd!1,c~i) -> Trash() kdegTSP1i0noUB

vegfr2(l1,CD47bd!1,dimer!2,c~i).vegfr2(l1,CD47bd,dimer!2,c~i).CD47SIRPa(TSP1bd!+,VEGFR2bd!1,c~i) -> Trash() kdegTSP1i0noUB

vegfr2(l1,CD47bd!1,dimer!3,c~i).vegfr2(l1,CD47bd!2,dimer!3,c~i).CD47SIRPa(TSP1bd!+,VEGFR2bd!1,c~i).CD47SIRPa(VEGFR2bd!2,c~i) -> Trash() kdegTSP1i0noUB

vegf(r!1,r!2,nrp1bd!+,c~i).vegfr2(l1!1,CD47bd!3,Y1175~pY,c~i).vegfr2(l1!2,CD47bd,c~i).CD47SIRPa(TSP1bd!+,VEGFR2bd!3,c~i) -> Trash() kdegTSP1NRP1i0

vegf(r!1,r!2,nrp1bd!+,c~i).vegfr2(l1!1,CD47bd,Y1175~pY,c~i).vegfr2(l1!2,CD47bd!3,c~i).CD47SIRPa(TSP1bd!+,VEGFR2bd!3,c~i) -> Trash() kdegTSP1NRP1i0

vegf(r!1,r!2,nrp1bd!+,c~i).vegfr2(l1!1,CD47bd!3,Y1175~pY,c~i).vegfr2(l1!2,CD47bd!4,c~i).CD47SIRPa(TSP1bd!+,VEGFR2bd!3,c~i). CD47SIRPa(VEGFR2bd!4,c~i) -> Trash() kdegTSP1NRP1i0

vegf(r!1,r!2,nrp1bd!+,c~i).vegfr2(l1!1,CD47bd!3,Y1175~Y,c~i).vegfr2(l1!2,CD47bd,Y1175~Y,c~i).CD47SIRPa(TSP1bd!+,VEGFR2bd!3,c~i) -> Trash() kdegTSP1NRP1i0noUB

**vegf(r!1,r!2,nrp1bd!+,c~i).vegfr2(l1!1,CD47bd!3,Y1175~Y,c~i).vegfr2(l1!2,CD47bd!4,Y1175~Y,c~i).CD47SIRPa(TSP1bd!+,VEGFR2bd!3,c~i).CD47SIRPa(VEGFR2bd!4,c~i) -> **

Trash() kdegTSP1NRP1i0noUB

# R2 receptor synthesis

I() -> I() + vegfr2(l1,Y1175~Y,CD47bd,dimer,c~s)   ksingleR2syn

vegfr2(l1,dimer,CD47bd!1,c~s).CD47SIRPa(TSP1bd,VEGFR2bd!1,c~s) -> vegfr2(l1,dimer,CD47bd!1,c~i).CD47SIRPa(TSP1bd,VEGFR2bd!1,c~i)   ksingleR2si

vegfr2(l1,dimer,CD47bd!1,c~s).CD47SIRPa(TSP1bd!+,VEGFR2bd!1,c~s) -> vegfr2(l1,dimer,CD47bd!1,c~i).CD47SIRPa(TSP1bd!+,VEGFR2bd!1,c~i)   ksingleR2TSP1si

**vegfr2(l1,Y1175~Y,CD47bd!1,dimer!3,c~s).CD47SIRPa(TSP1bd,VEGFR2bd!1,c~s).vegfr2(l1,Y1175~Y,CD47bd!2,dimer!3,c~s).CD47SIRPa(TSP1bd,VEGFR2bd!2,c~s) **

-> vegfr2(l1,Y1175~Y,CD47bd!1,dimer!3,c~i).CD47SIRPa(TSP1bd,VEGFR2bd!1,c~i).vegfr2(l1,Y1175~Y,CD47bd!2,dimer!3,c~i).CD47SIRPa(TSP1bd,VEGFR2bd!2,c~i) ksingleR2si

**vegfr2(l1,Y1175~Y,CD47bd!1,dimer!3,c~s).CD47SIRPa(TSP1bd!+,VEGFR2bd!1,c~s).vegfr2(l1,Y1175~Y,CD47bd!2,dimer!3,c~s).CD47SIRPa(TSP1bd!+,VEGFR2bd!2,c~s) **

-> vegfr2(l1,Y1175~Y,CD47bd!1,dimer!3,c~i).CD47SIRPa(TSP1bd!+,VEGFR2bd!1,c~i).vegfr2(l1,Y1175~Y,CD47bd!2,dimer!3,c~i).CD47SIRPa(TSP1bd!+,VEGFR2bd!2,c~i) ksingleR2TSP1si

```
vegfr2(l1,dimer,CD47bd,c~s) -> vegfr2(l1,dimer,CD47bd,c~i)   ksingleR2si

vegfr2(l1,CD47bd,dimer!3,c~s).vegfr2(l1,CD47bd,dimer!3,c~s) \

-> vegfr2(l1,CD47bd,dimer!3,c~i).vegfr2(l1,CD47bd,dimer!3,c~i) ksingleR2si



# Activating PLCgamma



PLCgamma(Yplc~Y) + vegfr2(Y1175~pY,c~s) -> PLCgamma(Yplc~pY) + vegfr2(Y1175~pY,c~s)
kpPLCgamma/(kmPLCgammaR2+yplcgamma)

PLCgamma(Yplc~Y) + vegfr2(Y1175~pY,c~i) -> PLCgamma(Yplc~pY) + vegfr2(Y1175~pY,c~i)
kpPLCgamma/(kmPLCgammaR2+yplcgamma)

PLCgamma(Yplc~pY) -> PLCgamma(Yplc~Y)     kdpPLCgamma



# IP3 and DAG generation

 PLCgamma(R2binding,Yplc~pY) + PI(PIsite~3P) -> IP3_cyto(ip3rbd) + PLCgamma(R2binding,Yplc~pY)
kcatPLCgammaDAG*freepip2^(nDAG-1)/(kmPIP2PLCgamma^nDAG+freepip2^nDAG)

 PLCgamma(R2binding,Yplc~pY) + PI(PIsite~3P) -> DAG(pkcbd) + PLCgamma(R2binding,Yplc~pY)
kcatPLCgammaDAG*freepip2^(nDAG-1)/(kmPIP2PLCgamma^nDAG+freepip2^nDAG)



I() -> I() + PI(PIsite~3P) kPIP2gen

IP3_cyto(ip3rbd) -> Trash()  kdeg_ip3

DAG(pkcbd) -> Trash()   kdeg_DAG



Calcium_cyto(bd) + CaF(cabd) <-> Calcium_cyto(bd!1).CaF(cabd!1) KBon,KBoff

I() -> I() + Istim()  ICrac*(Kcrac^ncrac/(Kcrac^ncrac+Caer^ncrac))/tau_stim-Iopenstim/tau_stim

I() -> I() + Calcium_cyto(bd)  (VolER/Volcyto)*Iip3Ramp*(Caer-
Cac)*(freeip3cyto^3.8/(freeip3cyto^3.8+KmIP3R^3.8))*(KiCa^3.8/(KiCa^3.8+Cac^3.8))

I() -> I() + CaER(bd)  -Iip3Ramp*(Caer-Cac)*(freeip3cyto^3.8/(freeip3cyto^3.8+KmIP3R^3.8))*(KiCa^3.8/(KiCa^3.8+Cac^3.8))*(
1/(1+CSQN_total/(KCSQN+Caer)^2) )

I() -> I() + Calcium_cyto(bd)   -I_PMCAbar*Cac^1.4/(KmPMCA^1.4+Cac^1.4) + Iopenstim  # PMCA pump

I() -> I() + Calcium_cyto(bd)   -I_ERCA*(Cac/(KmERCA+Cac))^2 + KleakER*(Caer-Cac)^2    # SERCA pump

I() -> I() + CaER(bd)  I_ERCA*(Cac/(KmERCA+Cac))^2*(Volcyto/VolER)*(1/(1+CSQN_total/(KCSQN+Caer)^2)) # SERCA pump

I() -> I() + CaER(bd)  -KleakER*(Volcyto/VolER)*((Caer-Cac)^2)*(1/(1+CSQN_total/(KCSQN+Caer)^2))
```

```
# Calcium binding to CaM

CaM(NCaM1) + Calcium_cyto(bd) <-> CaM(NCaM1!1).Calcium_cyto(bd!1)  konCaNCaM1,koffCaNCaM1

CaM(NCaM2) + Calcium_cyto(bd) <-> CaM(NCaM2!1).Calcium_cyto(bd!1)  konCaNCaM2,koffCaNCaM2

CaM(CCaM1) + Calcium_cyto(bd) <-> CaM(CCaM1!1).Calcium_cyto(bd!1)  konCaCCaM1,koffCaCCaM1

CaM(CCaM2) + Calcium_cyto(bd) <-> CaM(CCaM2!1).Calcium_cyto(bd!1)  konCaCCaM2,koffCaCCaM2



PKC(CalciumBD) + Calcium_cyto(bd) <-> PKC(CalciumBD!1).Calcium_cyto(bd!1) konCaPKC,koffCaPKC

PKC(DAGBD) + DAG(pkcbd) <-> PKC(DAGBD!1).DAG(pkcbd!1) konDAGPKC,koffDAGPKC


CIB1(EF1) + Calcium_cyto(bd) <->  CIB1(EF1!1).Calcium_cyto(bd!1)  kon1CaCIB1,koff1CaCIB1

CIB1(EF2) + Calcium_cyto(bd) <->  CIB1(EF2!1).Calcium_cyto(bd!1)  kon2CaCIB1,koff2CaCIB1


CIB1(EF1!+,EF2!+,sk1bd,location~cytosol) + SphK(CIB1bd,Serk~pS) <-> \

CIB1(EF1!+,EF2!+,sk1bd!1,location~cytosol).SphK(CIB1bd!1,Serk~pS) konCIB1SphK1,koffCIB1SphK1


ERK2(S2~pS) + SphK(Serk~S) -> ERK2(S2~pS) + SphK(Serk~pS)  kcatERK/(freeSphK1+kmERKSK1)


CIB1(EF1!+,EF2!+,sk1bd!+,location~cytosol) <-> CIB1(EF1!+,EF2!+,sk1bd!+,location~membrane)   ktSK1,ktoffSK1

CIB1(EF1,EF2,location~membrane) -> CIB1(EF1,EF2,location~cytosol)  koffSK1


PKC(CalciumBD!+,DAGBD!1).DAG(pkcbd!1) + Raf(Spkc~S) -> \

PKC(CalciumBD!+,DAGBD!1).DAG(pkcbd!1) + Raf(Spkc~pS) kcatPKC/(freeraf+kmPKCRaf)


SphK(Serk~pS) -> SphK(Serk~S) kdpSK1


Raf(Spkc~pS) -> Raf(Spkc~S) kdpPKCRaf


I() -> I() + Sph(skbd) kSphgen


CIB1(sk1bd!1,location~membrane).SphK(CIB1bd!1,Serk~pS) +  Sph(skbd) -> S1P(bd) +
CIB1(sk1bd!1,location~membrane).SphK(CIB1bd!1,Serk~pS)   \
```

kcatSK1Sph/(KmSK1Sph+freesphingosin)

S1P(bd) -> Sph(skbd)   kdpS1P

I() -> I() + RasGTP(rafbd) kS1PRas*frees1p/(KmS1PRas+frees1p)-kRasGAP*gtpfreeras

Raf(rasbd) + RasGTP(rafbd) <-> Raf(rasbd!1).RasGTP(rafbd!1) konRasRaf,koffRasRaf

Raf(rasbd!1,Y1Y2~Y).RasGTP(rafbd!1) -> Raf(rasbd!1,Y1Y2~pY).RasGTP(rafbd!1) kpRaf # Activation of Raf by Tyrosine phosphorylation

Raf(Y1Y2~pY) -> Raf(Y1Y2~Y) kdpRaf

MEK12(S1~S) + Raf(Y1Y2~pY,Spkc~S) -> Raf(Y1Y2~pY,Spkc~S) + MEK12(S1~pS) kpMEK12Raf1/(KmMEK12Raf+mek12s1)

MEK12(S2~S) + Raf(Y1Y2~pY,Spkc~S) -> Raf(Y1Y2~pY,Spkc~S) + MEK12(S2~pS) kpMEK12Raf2/(KmMEK12Raf+mek12s2)

MEK12(S1~S) + Raf(Y1Y2~pY,Spkc~pS) -> Raf(Y1Y2~pY,Spkc~pS) + MEK12(S1~pS) kpMEK12Raf1/(KmMEK12Raf+mek12s2)

MEK12(S2~S) + Raf(Y1Y2~pY,Spkc~pS) -> Raf(Y1Y2~pY,Spkc~pS) + MEK12(S2~pS) kpMEK12Raf2/(KmMEK12Raf+mek12s2)

MEK12(S1~S) + Raf(Y1Y2~Y,Spkc~pS) -> Raf(Y1Y2~Y,Spkc~pS) + MEK12(S1~pS) kpMEK12Raf1/(KmMEK12Raf+mek12s1)

MEK12(S2~S) + Raf(Y1Y2~Y,Spkc~pS) -> Raf(Y1Y2~Y,Spkc~pS) + MEK12(S2~pS) kpMEK12Raf2/(KmMEK12Raf+mek12s2)

MEK12(S1~pS) -> MEK12(S1~S) kdpMEK12_1

MEK12(S2~pS) -> MEK12(S2~S) kdpMEK12_2

MEK12(S1~pS,S2~pS) + ERK1(S1~S) -> MEK12(S1~pS,S2~pS) + ERK1(S1~pS) \

(kpMEK12ERK12_1/(kmMEKERK12+erk12s1))

MEK12(S1~pS,S2~pS) + ERK2(S2~S) -> \

MEK12(S1~pS,S2~pS) + ERK2(S2~pS) \

(kpMEK12ERK12_2/(kmMEKERK12+erk12s2))

ERK1(S1~pS) -> ERK1(S1~S) kdpERK12_1

ERK2(S2~pS) -> ERK2(S2~S) kdpERK12_2

TSP1 binding to CD47SIRPa

```
I() -> I() + TSP1(CD47bd)  -
(koffCD47TSP1/kDCD47TSP1)/fextmolar*tsp1frees*cellarea*cd47s+koffCD47TSP1*cd47tsp1s*cellarea*(1/fextmolar)

TSP1(CD47bd) + CD47SIRPa(TSP1bd,c~s) -> TSP1(CD47bd) + TSP1(CD47bd!1).CD47SIRPa(TSP1bd!1,c~s)
(koffCD47TSP1/kDCD47TSP1)

TSP1(CD47bd!1).CD47SIRPa(TSP1bd!1,c~s) -> CD47SIRPa(TSP1bd,c~s)   koffCD47TSP1



vegfr2(CD47bd,c~s) + CD47SIRPa(VEGFR2bd,c~s) -> \

vegfr2(CD47bd!1,c~s).CD47SIRPa(VEGFR2bd!1,c~s)  kcd47free_on




end reaction rules



end model



## actions ##

generate_network({overwrite=>1,max_agg=>10})

writeMexfile({atol=>1e-8,rtol=>1e-8,t_start=>0,t_end=>100000,n_steps=>10000,max_num_steps=>50000,sparse=>1,stiff=>1})

writeSBML({})
```

3 Supplementary references

Adams DJ & Hill MA. (2004). Potassium channels and membrane potential in the modulation of intracellular calcium in vascular endothelial cells. *Journal of cardiovascular electrophysiology* **15,** 598-610.

Bhalla US, Ram PT & Iyengar R. (2002). MAP kinase phosphatase as a locus of flexibility in a mitogen-activated protein kinase signaling network. *Science* **297,** 1018-1023.

Carter TD & Ogden D. (1997). Kinetics of Ca2+ release by InsP3 in pig single aortic endothelial cells: evidence for an inhibitory role of cytosolic Ca2+ in regulating hormonally evoked Ca2+ spikes. *The Journal of physiology* **504 (Pt 1),** 17-33.

De Young GW & Keizer J. (1992). A single-pool inositol 1,4,5-trisphosphate-receptor-based model for agonist-stimulated oscillations in Ca2+ concentration. *Proceedings of the National Academy of Sciences of the United States of America* **89,** 9895-9899.

Imoukhuede PI & Popel AS. (2011). Quantification and cell-to-cell variation of vascular endothelial growth factor receptors. *Experimental cell research* **317,** 955-965.

Imoukhuede PI & Popel AS. (2012). Expression of VEGF receptors on endothelial cells in mouse skeletal muscle. *PloS one* **7,** e44791.

Isenberg JS, Annis DS, Pendrak ML, Ptaszynska M, Frazier WA, Mosher DF & Roberts DD. (2009). Differential interactions of thrombospondin-1, -2, and -4 with CD47 and effects on cGMP signaling and ischemic injury responses. *The Journal of biological chemistry* **284,** 1116-1125.

Luik RM, Wang B, Prakriya M, Wu MM & Lewis RS. (2008). Oligomerization of STIM1 couples ER calcium depletion to CRAC channel activation. *Nature* **454,** 538-542.

Mac Gabhann F & Popel AS. (2007a). Dimerization of VEGF receptors and implications for signal transduction: a computational study. *Biophysical chemistry* **128,** 125-139.

Mac Gabhann F & Popel AS. (2007b). Interactions of VEGF isoforms with VEGFR-1, VEGFR-2, and neuropilin in vivo: a computational model of human skeletal muscle. *American journal of physiology Heart and circulatory physiology* **292,** H459-474.

Pepke S, Kinzer-Ursem T, Mihalas S & Kennedy MB. (2010). A dynamic model of interactions of Ca2+, calmodulin, and catalytic subunits of Ca2+/calmodulin-dependent protein kinase II. *PLoS computational biology* **6,** e1000675.

Schmeitz C, Hernandez-Vargas EA, Fliegert R, Guse AH & Meyer-Hermann M. (2013). A mathematical model of T lymphocyte calcium dynamics derived from single transmembrane protein properties. *Frontiers in immunology* **4,** 277.

Silva HS, Kapela A & Tsoukias NM. (2007). A mathematical model of plasma membrane electrophysiology and calcium dynamics in vascular endothelial cells. *American journal of physiology Cell physiology* **293,** C277-293.

Wiesner TF, Berk BC & Nerem RM. (1996). A mathematical model of cytosolic calcium dynamics in human umbilical vein endothelial cells. *The American journal of physiology* **270,** C1556-1569.

Winslow RL, Scollan DF, Holmes A, Yung CK, Zhang J & Jafri MS. (2000). Electrophysiological modeling of cardiac ventricular function: from cell to organ. *Annual review of biomedical engineering* **2,** 119-155.

Yamniuk AP, Nguyen LT, Hoang TT & Vogel HJ. (2004). Metal ion binding properties and conformational states of calcium- and integrin-binding protein. *Biochemistry* **43,** 2558-2568.

Zhang XY, Birtwistle MR & Gallo JM. (2014). A General Network Pharmacodynamic Model-Based Design Pipeline for Customized Cancer Therapy Applied to the VEGFR Pathway. *CPT: pharmacometrics & systems pharmacology* **3,** e92.
